# Tiliroside from *Lagopsis supina* Ameliorates Myocardial Ischemia Injury in Zebrafish by Activating the *kdr*-Mediated PI3K-Akt and MAPK Signaling Pathways

**DOI:** 10.3390/ijms26052313

**Published:** 2025-03-05

**Authors:** Yuqing Dong, Xiaoyi Xia, Miaoyunhuan Wang, Jiahao Yu, Lizhen Wang, Li Yang, Kechun Liu, Junwei He, Xiaobin Li

**Affiliations:** 1 Engineering Research Center of Zebrafish Models for Human Diseases and Drug Screening of Shandong Province, Biology Institute, Qilu University of Technology (Shandong Academy of Sciences), Jinan 250103, China; dongyuqing777@163.com (Y.D.); wangmiaoyunhuan@163.com (M.W.); yu_jiahao0423@163.com (J.Y.); wlzh1106@126.com (L.W.); hliukch@sdas.org (K.L.); 2 Research Center of Natural Resources of Chinese Medicinal Materials and Ethnic Medicine, Jiangxi University of Chinese Medicine, Nanchang 330004, China; xiaxiaoyi@jxutcm.edu.cn (X.X.); yangli07971@163.com (L.Y.)

**Keywords:** myocardial ischemia, *Lagopsis supina*, tiliroside, zebrafish, PI3K-Akt pathway, MAPK pathway

## Abstract

*Lagopsis supina* (Steph. ex. Willd.) Ikonn.-Gal., an ancient Chinese herbal medicine, is traditionally used to treat blood stasis diseases such as myocardial ischemia (MI). However, its pharmacodynamics substances of the anti-MI effect and their potential mechanisms remain unclear. This study aims to elucidate the pharmacodynamics effects of *L. supina* against MI and reveal their underlying mechanisms in zebrafish. LSD fraction was screened out for anti-MI active fraction from *L. supina* by isoprenaline hydrochloride (ISO)-induced zebrafish. It could increase the stroke volume, ejection fraction, and ventricular short-axis systolic rate in the zebrafish model. A total of 30 compounds (Nos. **1**–**30**) were isolated and identified from LSD by various chromatographic techniques and nuclear magnetic resonance spectroscopy. Among them, six compounds, including three lignin compounds (Nos. **15**, **16**, and **18**) and three flavonoid glycosides (Nos. **14**, **25**, and **26**), showed noticeable anti-MI activities, and tiliroside (No. **25**) was more active. Molecular docking indicated that tiliroside has a strong binding ability with the proteins KDR, PI3K, Akt, Erk, p38, Bcl-2, Bax, and Caspase3. In the end, the results of RT-qPCR manifested that tiliroside markedly upregulated expression levels of genes *kdr*, *pik3cb*, *akt2*, *mapk1*, *mapk11*, *mapk14*, and *bcl-2b* and prominently downregulated expression levels of genes *bax* and *caspase3*. According to the above results, tiliroside activated the *kdr*-mediated PI3K-Akt and MAPK signaling pathways to exert the anti-MI activity. These discoveries give a scientific basis for applying *L. supina* in MI treatment and suggest new avenues for developing tiliroside as a candidate for MI therapy.

## 1. Introduction

Myocardial ischemia (MI) is caused by insufficient blood and oxygen supply to the heart due to coronary artery stenosis, spasm, or embolism. In traditional Chinese medicine (TCM), MI is treated by restoring the body′s blood supply to the heart [1]. According to modern research, ischemia of the heart can cause myocardial cell apoptosis, leading to heart failure and decreased functionality [2]. The therapeutic effects of MI drugs are limited in many aspects, such as adverse reactions to the drug and interference by other medications [3]. Apoptosis exacerbates the symptoms of MI, so preventing apoptosis is crucial in treating MI [2,4]. In recent years, several signaling pathways, such as the PI3K-Akt, MAPK, Integrin/FAK, TNF-α/NF-κB, GPCR, and Hippo pathways, have been demonstrated to regulate apoptosis [5]. Furthermore, the PI3K-Akt and MAPK signaling pathways have been shown to regulate apoptosis caused by MI [5]. Moreover, some drugs, such as Guizhi-Fuling [6], bregenin [7], and L-theanine [8], can inhibit cell apoptosis to treat MI. KDR is a cell surface receptor that regulates cell survival, metabolism, and cell migration and can influence the PI3K-Akt and MAPK signaling pathways [9,10]. Therefore, the inhibition of *kdr*-mediated PI3K-Akt and MAPK signaling pathways and the suppression of apoptosis are important strategies for discovering MI therapeutic drugs.

In the process of drug development, an ideal experimental model is crucial for activity evaluation and mechanism research. The zebrafish model is characterized by fast reproduction, transparent embryos, low sample consumption, and high homology with the human genome [11]. It has been used for high-throughput screening of drugs, evaluation of drug efficacy, and mechanism research, particularly for insignificant amounts of natural products [12,13,14]. The ISO-induced zebrafish model has been widely used for evaluating anti-MI activity [15,16].

*Lagopsis supina* (Steph. ex. Willd.) Ikonn.-Gal., a perennial herb from the Lamiaceae family, is commonly found throughout China [17]. It is characterized by a spicy, slightly bitter taste and a cold nature, targeting the liver meridian [18]. This plant, as a Chinese herbal medicine, was first mentioned in “Shennong′s Herbal Classics” (神农本草经 in Chinese). “Shennong′s Herbal Classics” and the “Compendium of Materia Medica” (本草纲目 in Chinese) recorded that *L. supina* was used principally for treating blood stasis diseases such as MI and thrombosis due to its effects of promoting blood circulation and nourishing blood [19,20,21]. In addition, other ancient Chinese books also record the therapeutic effects of *L. supina* on diseases such as heat in the blood (“Mingyi Bielu”), postpartum hemorrhage (“Arcane Essentials from the Imperial Library”), and poor blood circulation (“Compendium of Materia Medica”). Our team has been conducting systematic modern pharmacological research on the traditional application of *L. supina* for a long time. Our previous studies also demonstrated that the ethanol extract of *L. supina* (LS) can promote blood circulation to prevent blood stasis and is antithrombotic, anti-inflammatory, and diuretic [21,22,23,24,25]. Especially, a 60% ethanol–water extract from the macroporous adsorbed resin (LSD) of LS exhibited a significant anti-MI effect in isoproterenol (ISO)-induced rats, identifying it as the significant effective fraction [20]. However, the bioactive constituents within this fraction and their potential mechanisms remain unclear.

**Figure 1:**
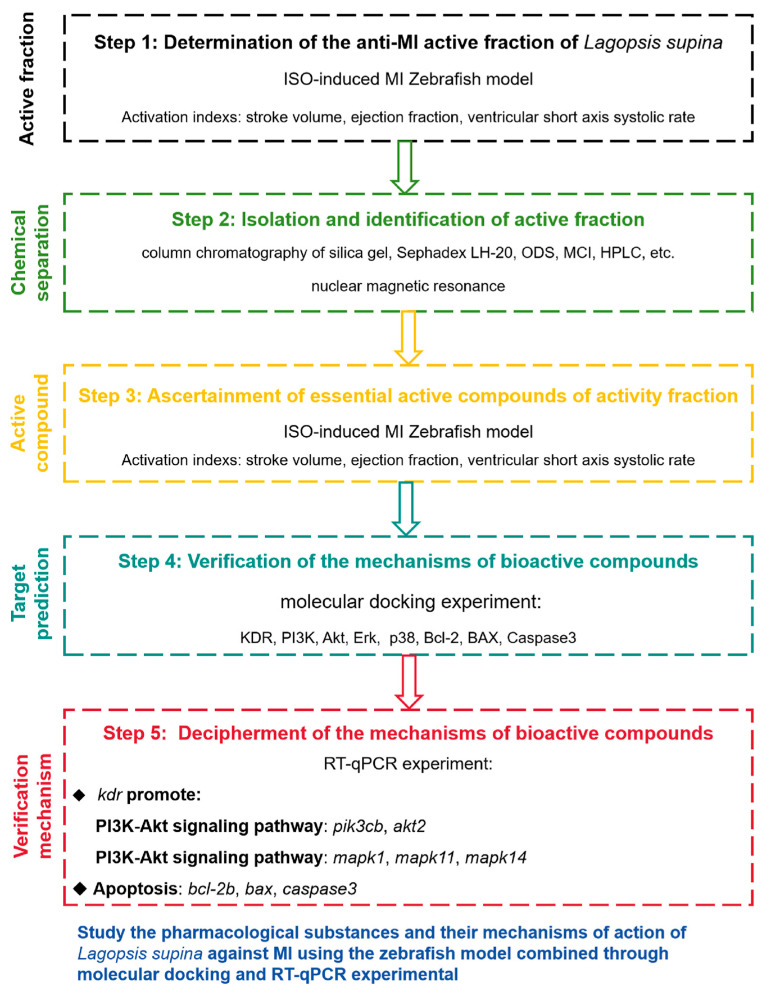
Experimental design process.

In this article, we systematically isolated and purified the anti-MI active fraction LSD and further revealed the specific pharmacodynamic material basis and potential mechanisms of active compound. The present work proceeded according to the following strategy (Figure 1): (1) Screening the active fraction of *L. supina* responsible for the anti-MI effects via an ISO-induced zebrafish model; (2) isolating and identifying the chemical constituents of the LSD fraction; (3) screening the compounds with anti-MI effects in an ISO-induced zebrafish model; (4) examining the binding interactions of active compounds with their possible targets through molecular docking; and (5) validating the potential mechanisms of bioactive compounds via RT-qPCR. This is the first systematic study of the effective constituents from *L. supina* with anti-MI activity and its potential action mechanisms. This research provides scientific evidence for the clinical application of this herb in treating MI and may lead to new candidate molecules for MI treatment.

## 2. Results

### 2.1. Anti-MI Activity of Extracts

**Figure 2:**
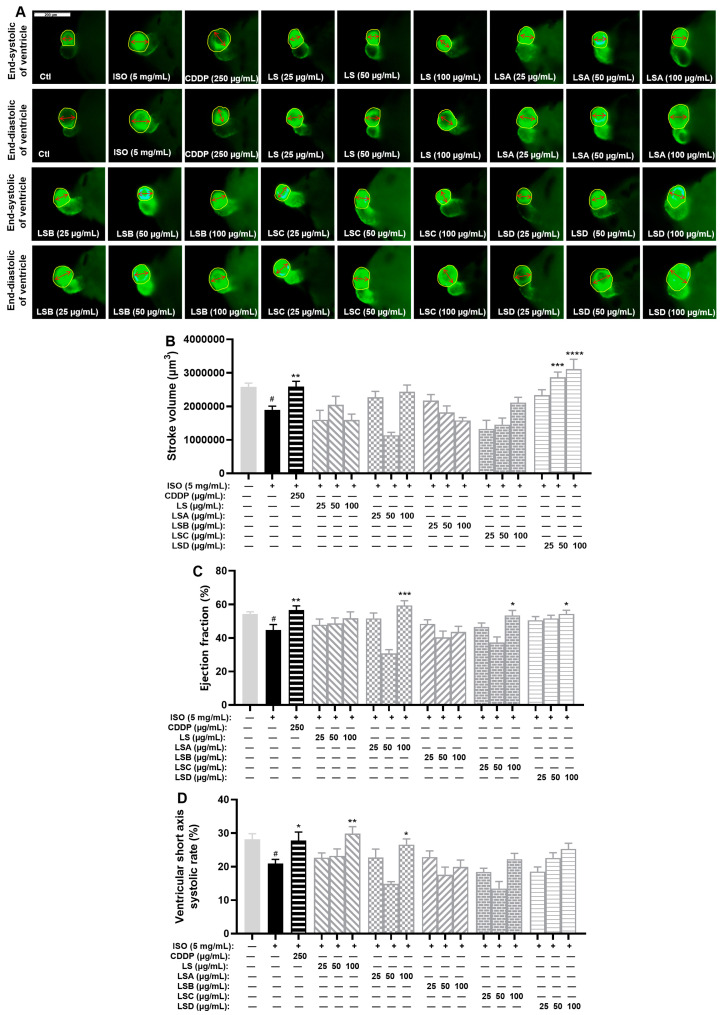
Results of anti-MI activity evaluation for four fractions (LSA-D) in zebrafish (*n* = 6–10). (**A**) Representative images of the end-systolic and end-diastolic regions of zebrafish ventricles in each group (yellow represents the ventricular region and red represents the length of the ventricular short axis). (**B**,**C**) Effects of LS and its four fractions (LSA-D) on stroke volume (**B**), ejection fraction (**C**), and ventricular short-axis systolic rate (**D**). The data are expressed as the means ± SEMs. ^#^ *p* < 0.05 vs. the C group; * *p* < 0.05, ** *p* < 0.01, *** *p* < 0.001, and **** *p* < 0.0001 vs. the ISO group.

In earlier research, we acquired the ethanol crude extract of *L. supina* (LS) along with its four fractions (LSA–D). The effects of anti-MI agents were measured by assessing the stroke volume, ejection fraction, and ventricular short-axis systolic rate of zebrafish after treatment with these samples. As shown in Figure 2, the stroke volume, ejection fraction, and ventricular short-axis systolic rate of zebrafish in the ISO-treated model group had significantly lower levels compared to the control group (*p* < 0.05). Compared with those in the model group, these three indices markedly increased in the Compound Danshen Dropping Pills (CDDP)-treated positive control group (*p* < 0.01, *p* < 0.01, and *p* < 0.05). Furthermore, compared with the model group, all the LS groups (25, 50, and 100 µg/mL) had various degrees of recovery effects on the ejection fraction and ventricular short-axis systolic rate, and the LSD group treated with 100 µg/mL LS had a therapeutic effect on the stroke volume and ejection fraction (Figure 2). Compared with the model group, the LSD group had a significantly greater stroke volume (*p* < 0.01 or *p* < 0.0001) and ejection fraction (*p* < 0.05) at concentrations above 25 µg/mL.

These results demonstrated that LS and LSD have evident anti-MI activity, which can improve the stroke volume, ejection fraction, or ventricular short-axis systolic rate in the ISO-induced zebrafish model.

### 2.2. Isolated Compounds from the LSD Fraction

**Figure 3:**
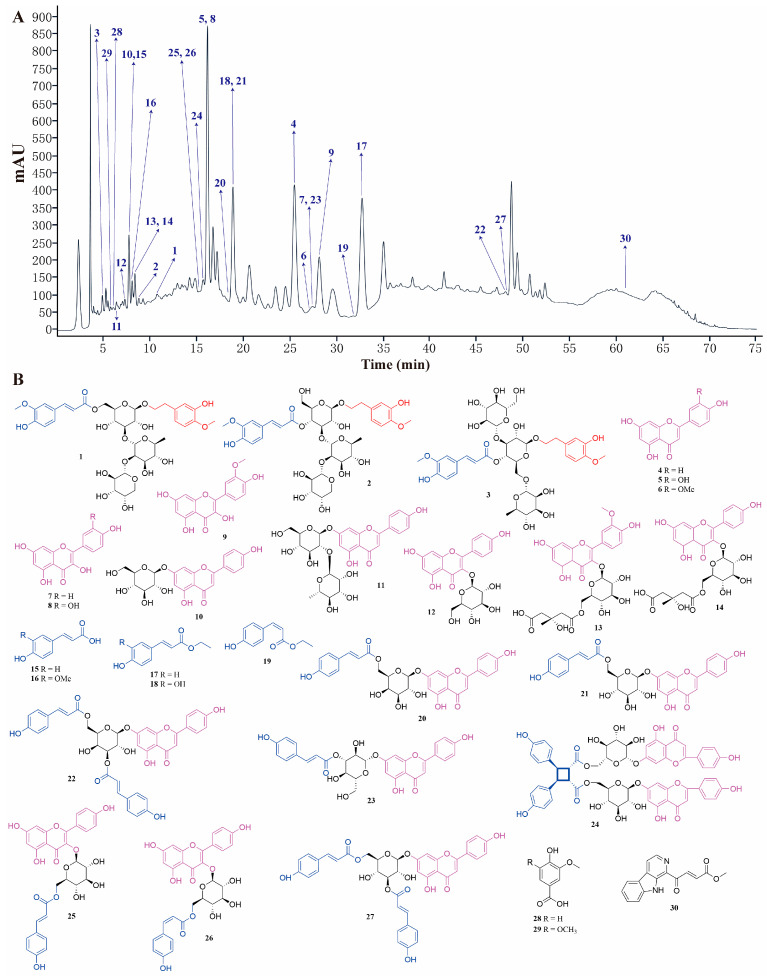
Thirty compounds were separated from LSD. (**A**) HPLC composition analysis chromatogram of LSD. (**B**) Structural diagram of the 30 compounds.

To elucidate the bioactive phytochemicals of the LSD fraction responsible for its anti-MI effect, we employed various chromatographic techniques, including silica gel column chromatography (CC), Sephadex LH-20 CC, octadecylsilyl (ODS) CC, MCI CC, and preparative HPLC. As a result, 30 phytochemicals (Nos. **1**–**30**) were isolated from the LSD fraction. The peak identification of each compound in HPLC and its corresponding chemical structure are presented in Figure 3. These structures were identified as Number **3** (No. **1**, [26]), stachysoside D (No. **2**, [27]), artselaeroside B (No. **3**, [28]), apigenin (No. **4**, [29]), luteolin (No. **5**, [29]), chrysoeriol (No. **6**, [30]), kaempferol (No. **7**, [31]), quercetin (No. **8**, [29]), isorhamnetin (No. **9**, [32]), apigenin-7-O-*β*-D-glucopyranoside (No. **10**, [29]), rhoifolin (No. **11**, [29]), kaempferol-3-O-*β*-D-glucopyranoside (No. **12**, [33]), isorhamnetin-3-O-*β*-D-[6″-(3-hydroxy-3-methylglutaryl)]-O-*β*-D-glucoside (No. **13**, [34]), kaempferol-3-O-*β*-D-glucopyranoside-6″-(3-hydroxy-3-methylglutarate) (No. **14**, [35]), *p*-coumaric acid (No. **15**, [36]), ferulic acid (No. **16**, [37]), *cis*-*p*-hydroxyl ethyl cinnamate (No. **17**, [38]), caffeic acid ethylester (No. **18**, [39]), *trans*-*p*-hydroxyl ethyl cinnamate (No. **19**, [39]), apigenin-7-O-(6″-(*E*)-p-coumaroyl)-*β*-D-galactopyranoside (No. **20**, [27]), apigenin-7-O-(6″-*E*-p-coumaroyl)-*β*-D-glucopyranoside (No. **21**, [40,41]), apigenin-7-O-(3″,6″-di-(*E*)-p-coumaroyl)-*β*-D-galactopyranoside (No. **22**, [42]), apigenin-7-O-(3′′-p-coumaryl)-glucoside (No. **23**, [43]), palhinoside A (No. **24**, [35]), tiliroside (No. **25**, [44]), *cis*-tiliroside (No. **26**, [44]), anisofolin A (No. **27**, [45]), vanillic acid (No. **28**, [46]), syringic acid (No. **29**, [47]), and 4-(9H-B-carbolin-1-yl)-4-oxobut-2-enoic acid methyl ester (No. **30**, [48]), by comparing their NMR data with those reported earlier.

**No. 1** (compound 3): Yellow powder. ^1^H NMR (400 MHz, CD_3_OD-*d*_4_): δ_H_ 7.62 (1H, d, *J* = 15.9 Hz, H-7′), 7.16 (1H, d, *J* = 1.8 Hz, H-2′), 7.02 (1H, dd, *J* = 8.2, 1.8 Hz, H-6′), 6.80 (1H, d, *J* = 8.2 Hz, H-5′), 6.69 (1H, d, *J* = 2.0 Hz, H-2), 6.66 (1H, d, *J* = 8.2 Hz, H-5), 6.61 (1H, dd, *J* = 8.2, 2.0 Hz, H-6), 6.39 (1H, d, *J* = 15.9 Hz, H-8′), 5.48 (1H, d, *J* = 1.2 Hz, H-1‴), 4.49 (1H, d, *J* = 2.0 Hz, H-6″), 4.39 (1H, d, *J* = 6.3 Hz, H-6″), 4.36 (1H, d, *J* = 2.3 Hz, H-1″″), 4.33 (1H, d, *J* = 7.9 Hz, H-1″), 3.98 (1H, m, H-2‴), 3.94 (1H, d, *J* = 9.1 Hz, H-7), 3.86 (3H, s, 3′-OCH_3_), 3.85 (1H, m, H-5″″), 3.79 (1H, m, H-3‴), 3.77 (1H, m, H-4″″), 3.75 (3H, s, 4-OCH_3_), 3.73 (1H, dd, *J* = 7.5, 1.7 Hz, H-7), 3.63 (1H, m, H-2″″), 3.55 (2H, m, H-5′, 5‴), 3.52 (1H, m, H-5″″), 3.51 (1H, d, *J* = 4.2 Hz, H-3″″), 3.50 (1H, d, *J* = 4.0 Hz, H-3″), 3.40 (2H, m, H-4″), 4‴), 3.32 (1H, m, H-2″), 2.80 (2H, t, *J* = 7.6 Hz, H-8), and 1.24 (1H, d, *J* = 6.2 Hz, H-6‴). ^13^C NMR (100 MHz, CD_3_OD-*d*_4_): δ_C_ 169.2 (C-9′), 150.8 (C-3′), 149.5 (C-4′), 147.6 (C-7′), 147.5 (C-3), 147.3 (C-4), 132.9 (C-1), 127.8 (C-1′), 124.4 (C-6′), 121.3 (C-6), 117.2 (C-5), 116.6 (C-5′), 115.4 (C-8′), 112.9 (C-2), 111.8 (C-2′), 107.6 (C-1″″), 104.5 (C-1″), 101.8 (C-1‴), 84.5 (C-3″), 82.7 (C-2‴), 75.7 (C-2″), 75.5 (C-5″), 74.6 (C-4‴, 3″″), 73.0 (C-2″″), 72.5 (C-7), 72.3 (C-3‴), 70.7 (C-5‴), 70.1 (C-4″), 70.0 (C-4″″), 67.5 (C-5″″), 64.8 (C-6″), 56.6 (4, 3′-OCH_3_), 36.9 (C-8), and 18.1 (C-6‴).

**No. 2** (stachysoside D): Yellow powder. ^1^H NMR (600 MHz, DMSO-*d*_6_): δ_H_ 7.54 (1H, d, *J* = 15.9 Hz, H-7′), 7.30 (1H, d, *J* = 1.5 Hz, H-2′), 7.10 (1H, dd, *J* = 8.2, 1.5 Hz, H-6′), 6.78–6.82 (2H, m, H-5, 5′), 6.68 (1H, d, *J* = 2.0 Hz, H-2), 6.63 (1H, dd, *J* = 8.2, 2.0 Hz, H-6), 6.43 (1H, d, *J* = 15.9 Hz, H-8′), 5.26 (1H, s, H-1‴), 4.73 (1H, m, H-4″), 4.38 (1H, d, *J* = 7.8 Hz, H-1″), 4.21 (1H, d, *J* = 6.7 Hz, H-1″″), 3.91 (1H, m, H-8), 3.80 (3H, s, 3′-OCH_3_), 3.77 (1H, m, H-2‴), 3.72 (3H, s, 4-OCH_3_), 3.67 (1H, m, H-3″), 3.64 (1H, m, H-5″″), 3.61 (1H, m, H-8), 3.58 (1H, m, H-4″″), 3.47 (1H, m, H-5′′), 3.41 (1H, m, H-6″), 3.39 (1H, m, H-5‴), 3.38 (1H, m, H-5″″), 3.36 (1H, m, H-6″), 3.31–3.34 (3H, m, H-3‴, 2″″, 3″″), 3.22 (1H, m, H-2′′), 3.07 (1H, t, *J* = 9.6 Hz, H-4‴), 2.73 (2H, m, H-7), and 0.96 (3H, d, *J* = 6.2 Hz, H-6‴). ^13^C NMR (150 MHz, DMSO-*d*_6_): δ_C_ 165.9 (C-9′), 149.5 (C-4′), 148.0 (C-3′), 146.3 (C-3), 146.1 (C-4), 145.7 (C-7′), 131.0 (C-1), 125.6 (C-1′), 123.3 (C-6′), 119.4 (C-6), 116.3 (C-2), 115.5 (C-5, 5′), 114.1 (C-8′), 111.1 (C-2′), 106.0 (C-1″″), 102.3 (C-1″), 100.1 (C-1‴), 81.0 (C-2‴), 80.1 (C-3″), 74.5 (C-2″), 74.3 (C-5″), 72.6 (C-3″″), 72.3 (C-4‴), 71.2 (C-2″″), 70.4 (C-3‴), 70.2 (C-8), 69.0 (C-4″), 68.6 (C-5‴), 67.8 (C-4″″), 65.8 (C-5″″), 60.7 (C-6′′), 55.7 (4, 3′-OCH_3_), 35.0 (C-7), and 18.1 (C-6‴). 

**No. 3** (artselaeroside B): White powder. ^1^H NMR (400 MHz, DMSO-*d*_6_): δ_H_ 7.55 (1H, d, *J* = 15.8 Hz, H-7′), 7.29 (1H, d, *J* = 1.7 Hz, H-2′), 7.10 (1H, dd, *J* = 8.1, 1.7 Hz, H-6′), 6.79–6.81 (2H, m, H-5, 5′), 6.69 (1H, d, *J* = 2.0 Hz, H-2), 6.65 (1H, dd, *J* = 8.0, 2.0 Hz, H-6), 6.42 (1H, d, *J* = 15.9 Hz, H-8′), 5.03 (1H, s, H-1″″), 4.38 (1H, d, *J* = 7.9 Hz, H-1″), 4.17 (1H, d, *J* = 7.8 Hz, H-1‴), 3.80 (3H, s, 3′-OCH_3_), 3.72 (3H, s, 4-OCH_3_), and 0.97 (3H, d, *J* = 6.1 Hz, H-6‴). ^13^C NMR (100 MHz, DMSO-*d*_6_): δ_C_ 166.1 (C-9′), 149.6 (C-3′), 147.9 (C-4′), 146.3 (C-4), 146.1 (C-3), 145.9 (C-7′), 131.1 (C-1), 125.5 (C-1′), 123.3 (C-6′), 119.4 (C-6), 116.4 (C-5), 115.6 (C-8′), 113.9 (C-5′), 112.3 (C-2), 111.1 (C-2′), 103.4 (C-1‴), 102.2 (C-1″), 101.3 (C-1″″), 79.0 (C-3″), 76.9 (C-3‴), 76.5 (C-5‴), 74.4 (C-2″), 73.5 (C-2‴), 73.2 (C-5″), 71.7 (C-4″″), 70.5 (C-3″″), 70.4 (C-2″″), 70.2 (C-8), 69.9 (C-4‴), 69.1 (C-4″), 68.8 (C-5″″), 68.1 (C-6″), 61.0 (C-6‴), 55.7 and 55.6 (4, 3′-OCH_3_), 35.0 (C-7), and 18.1 (C-6″″).

**No. 4** (apigenin): Yellow powder. ^1^H NMR (600 MHz, DMSO-*d*_6_): δ_H_ 12.96 (1H, s, 5-OH), 7.91 (2H, d, *J* = 8.8 Hz, H-2′, 6′), 6.91 (2H, d, *J* = 8.8 Hz, H-3′, 5′), 6.76 (1H, s, H-3), 6.47 (1H, d, *J* = 1.9 Hz, H-8), and 6.18 (1H, d, *J* = 2.1 Hz, H-6). ^13^C NMR (150 MHz, DMSO-*d*_6_): δ_C_ 181.7 (C-4), 164.1 (C-2), 163.7 (C-7), 161.5 (C-9), 161.2 (C-4′), 157.3 (C-5), 128.5 (C-2′, 6′), 121.2 (C-1′), 116.0 (C-3′, 5′), 103.7 (C-10), 102.8 (C-3), 98.9 (C-6), and 94.0 (C-8).

**No. 5** (luteolin): Yellow powder. ^1^H NMR (600 MHz, DMSO-*d*_6_): δ_H_ 12.96 (1H, s, 5-OH), 7.38–7.40 (2H, m, H-2′, 6′), 6.88 (1H, d, *J* = 8.3 Hz, H-5′), 6.64 (1H, s, H-3), 6.44 (1H, s, H-8), and 6.17 (1H, s, H-6). ^13^C NMR (150 MHz, DMSO-*d*_6_): δ_C_ 181.6 (C-4), 164.6 (C-7), 163.9 (C-2), 161.5 (C-5), 157.3 (C-9), 149.9 (C-4′), 145.8 (C-3′), 121.3 (C-1′), 118.9 (C-6′), 116.0 (C-5′), 113.3 (C-2′), 103.5 (C-10), 102.7 (C-3), 98.9 (C-6), and 93.9 (C-8).

**No. 6** (chrysoeriol): Yellow powder. ^1^H NMR (600 MHz, DMSO-*d*_6_): δ_H_ 12.96 (1H, s, 5-OH), 7.53–7.55 (2H, m, H-2′, 6′), 6.92 (1H, d, *J* = 8.3 Hz, H-5′), 6.85 (1H, s, H-3), 6.45 (1H, s, H-8), 6.14 (1H, s, H-6), and 3.88 (3H, s, 3′-OCH_3_). ^13^C NMR (150 MHz, DMSO-*d*_6_): δ_C_ 181.5 (C-4), 164.6 (C-7), 163.4 (C-2), 161.4 (C-5), 157.4 (C-9), 150.9 (C-4′), 148.0 (C-3′), 120.3 (C-6′), 115.8 (C-5′), 110.1 (C-2′), 103.0 (C-3), 99.1 (C-6), 94.2 (C-8), and 55.9 (3′-OCH_3_).

**No. 7** (kaempferol): Yellow crystal (methanol). ^1^H NMR (600 MHz, DMSO-*d*_6_): δ_H_ 12.48 (1H, s, 5-OH), 8.04 (2H, d, *J* = 8.9 Hz, H-2′, 6′), 6.92 (1H, d, *J* = 8.9 Hz, H-3′,5′), 6.43 (1H, d, *J* = 1.8 Hz, H-8), and 6.19 (1H, d, *J* = 1.8 Hz, H-6). ^13^C NMR (150 MHz, DMSO-*d*_6_): δC 175.9 (C-4), 164.0 (C-7), 160.7 (C-5), 159.2 (C-4′), 156.2 (C-9), 146.8 (C-2), 135.7 (C-3), 129.5 (C-2′,6′), 121.7 (C-1′), 115.5 (C-3′,5′), 103.1 (C-10), 98.3 (C-6), and 93.5 (C-8).

**No. 8** (quercetin): Yellow powder. ^1^H NMR (600 MHz, DMSO-*d*_6_): δ_H_ 12.48 (1H, s, 5-OH), 7.67 (1H, d, *J* = 2.1 Hz, H-2′), 7.54 (1H, dd, *J* = 8.5, 2.1 Hz, H-6′), 6.86 (1H, d, *J* = 8.5 Hz, H-5′), 6.40 (1H, d, *J* = 1.8 Hz, H-8), and 6.17 (1H, d, *J* = 1.8 Hz, H-6). ^13^C NMR (150 MHz, DMSO-*d*_6_): δ_C_ 178.5 (C-4), 164.2 (C-7), 160.7 (C-5), 156.1 (C-9), 147.7 (C-4′), 146.7 (C-2), 145.1 (C-3′), 135.8 (C-3), 122.0 (C-1′), 119.9 (C-6′), 115.6 (C-2′), 115.0 (C-5′), 102.9 (C-10), 98.3 (C-6), and 93.4 (C-8).

**No. 9** (isorhamnetin): Yellow powder. ^1^H NMR (600 MHz, DMSO-*d*_6_): δ_H_ 12.46 (1H, s, 5-OH), 7.75 (1H, d, *J* = 1.7 Hz, H-2′), 7.68 (1H, dd, *J* = 8.4, 1.7 Hz, H-6′), 6.93 (1H, d, *J* = 8.4 Hz, H-5′), 6.47 (1H, d, *J* = 1.9 Hz, H-8), 6.19 (1H, d, *J* = 1.9 Hz, H-6), and 3.84 (3H, s, 3′-OCH_3_). ^13^C NMR (150 MHz, DMSO-*d*_6_): δ_C_ 175.9 (C-4), 164.0 (C-7), 160.7 (C-5), 156.1 (C-9), 148.8 (C-3′), 147.3 (C-2), 146.6 (C-4′), 122.0 (C-1′), 121.7 (C-6′), 115.5 (C-5′), 111.7 (C-2′), 103.0 (C-10), 98.2 (C-6), 93.6 (C-8), and 55.8 (3′-OCH_3_).

**No. 10** (apigenin-7-O-*β*-D-glucopyranoside): Yellow powder. ^1^H NMR (600 MHz, DMSO-*d*_6_): δ_H_ 12.97 (1H, s, 5-OH), 7.94 (2H, d, *J* = 8.8 Hz, H-2′, 6′), 6.93 (1H, d, *J* = 8.8 Hz, H-3′,5′), 6.86 (1H, s, H-3), 6.83 (1H, d, *J* = 2.0 Hz, H-8), 6.44 (1H, d, *J* = 2.0 Hz, H-6), 5.07 (1H, d, *J* = 7.5 Hz, H-1″), and 3.19–3.72 (6H, H-2″, 3″, 4″, 5″, and 6″). ^13^C NMR (150 MHz, DMSO-*d*_6_): δ_C_ 182.0 (C-4), 164.3 (C-7), 162.9 (C-2), 161.5 (C-9), 161.1 (C-4′), 156.9 (C-5), 128.6 (C-2′,6′), 120.9 (C-1′), 116.0 (C-3′,5′), 105.3 (C-10), 103.1 (C-3), 99.9 (C-1″), 99.5 (C-6), 94.8 (C-8), 77.2 (C-5″), 76.4 (C-3″), 73.1 (C-2″), 69.5 (C-4″), and 60.6 (C-6″).

**No. 11** (rhoifolin): Yellow powder. ^1^H NMR (600 MHz, DMSO-*d*_6_): δ_H_ 12.97 (1H, s, 5-OH), 7.94 (2H, d, *J* = 8.7 Hz, H-2′, 6′), 6.94 (1H, d, *J* = 8.7 Hz, H-3′,5′), 6.86 (1H, s, H-3), 6.78 (1H, d, *J* = 1.4 Hz, H-8), 6.38 (1H, d, *J* = 1.4 Hz, H-6), 5.23 (1H, d, *J* = 7.4 Hz, H-1″), 5.14 (1H, s, H-1‴), and 1.21 (3H, d, *J* = 8.2 Hz, 6‴-CH3). ^13^C NMR (150 MHz, DMSO-*d*_6_): δ_C_ 182.0 (C-4), 164.3 (C-2), 162.6 (C-7), 161.5 (C-9), 161.1 (C-4′), 157.0 (C-5), 128.6 (C-2′, 6′), 121.0 (C-1′), 116.1 (C-3′, 5′), 105.5 (C-10), 103.2 (C-3), 100.6 (C-1″), 99.4 (C-6), 97.9 (C-1‴), 94.6 (C-8), 77.3 (C-2″), 77.1 (C-5″), 76.3 (C-3″), 71.9 (C-4‴), 70.5 (C-3‴), 70.4 (C-2‴), 69.7 (C-4″), 68.4 (C-5‴), 60.5 (C-6″), and 18.1 (C-6‴).

**No. 12** (kaempferol-3-O-*β*-D-glucopyranoside): Yellow powder. ^1^H NMR (600 MHz, DMSO-*d*_6_): δ_H_ 12.61 (1H, s, 5-OH), 8.04 (2H, d, *J* = 8.7 Hz, H-2′, 6′), 6.89 (1H, d, *J* = 8.7 Hz, H-3′,5′), 6.42 (1H, s, H-8), 6.20 (1H, s, H-6), 5.46 (1H, d, *J* = 7.6 Hz, H-1″), 3.57 (1H, br.d, *J* = 11.5 Hz, H-6″), 3.34 (1H, m, H-6″), 3.22 (1H, m, H-3″), 3.19 (1H, m, H-2″), and 3.08–3.12 (2H, m, H-4″) and H-5″). ^13^C NMR (150 MHz, DMSO-*d*_6_): δ_C_ 177.4 (C-4), 164.8 (C-7), 161.3 (C-5), 160.1 (C-4′), 156.5 (C-9), 156.2 (C-2), 133.2 (C-3), 130.9 (C-2′, 6′), 121.0 (C-1′), 115.2 (C-3′, 5′), 103.9 (C-10), 101.0 (C-1″), 98.9 (C-6), 93.8 (C-8), 77.5 (C-5″), 76.5 (C-3″), 74.3 (C-2″), 69.9 (C-4″), and 60.9 (C-6″).

**No. 13** (isorhamnetin-3-O-*β*-D-[6″)-(3-hydroxy-3-methylglutaryl)]-O-*β*-D-glucoside): Yellow powder. ^1^H NMR (400 MHz, DMSO-*d*_6_): δ_H_ 12.62 (1H, s, 5-OH), 7.81 (1H, d, *J* = 1.8 Hz, H-2′), 7.54 (1H, dd, *J* = 8.4, 1.8 Hz, H-6′), 6.92 (1H, d, *J* = 8.4 Hz, H-5′), 6.45 (1H, br.s, H-8), 6.21 (1H, br.s, H-6), 5.47 (1H, d, *J* = 7.2 Hz, H-1″), 4.11 (1H, br.d, *J* = 12.0 Hz, H-6″), 3.84 (3H, s, 3′-OCH_3_), 3.99 (1H, dd, *J* = 12.0, 6.5 Hz, H-6″), 3.33 (1H, m, H-3″), 3.21–3.26 (2H, m, H-2″) and H-5″), 3.10 (1H, m, H-4″), 2.28–2.38 (2H, m, H-4‴), 2.23–2.54 (4H, m, H-2‴, 4‴), 2.15–2.25 (1H, m, H-2‴), and 0.99 (3H, s, 6‴-CH_3_). ^13^C NMR (100 MHz, DMSO-*d*_6_): δ_C_ 177.3 (C-4), 173.6 (C-1‴), 170.3 (C-5‴), 164.6 (C-7), 161.2 (C-5), 156.4 (C-9), 149.7 (C-3′), 147.0 (C-4′), 132.9 (C-3), 122.4 (C-1′), 120.9 (C-6′), 115.3 (C-5′), 113.2 (C-2′), 103.9 (C-10), 101.0 (C-1″), 98.9 (C-6), 93.9 (C-8), 76.1 (C-3″), 74.2 (C-5″), 74.1 (C-2″), 70.0 (C-4″), 68.8 (C-3‴), 63.0 (C-6″), 55.7 (3′-OCH3), 45.5 (C-2‴), 45.4 (C-4‴), and 27.2 (C-6‴).

**No. 14** (kaempferol-3-O-*β*-D-glucopyranoside-6″)-(3-hydroxy-3-methylglutarate): Yellow powder. ^1^H NMR (600 MHz, DMSO-*d*_6_): δ_H_ 12.57 (1H, s, 5-OH), 7.99 (2H, d, *J* = 8.9 Hz, H-2′, 6′), 6.87 (2H, d, *J* = 8.9 Hz, H-3′, 5′), 6.43 (1H, d, *J* = 1.9 Hz, H-8), 6.21 (1H, d, *J* = 1.9 Hz, H-6), 5.39 (1H, d, *J* = 7.4 Hz, H-1″), 4.14 (1H, br.d, *J* = 11.9 Hz, H-6″), 3.92 (1H, dd, *J* = 11.9, 6.6 Hz, H-6″), 3.32 (1H, m, H-3″), 3.17–3.26 (2H, m, H-2″, 5″), 3.11 (1H, m, H-4″), 2.42 (1H, d, *J* = 14.1 Hz, H-2‴), 2.29–2.33 (3H, m, H-2‴, 4‴), and 1.05 (3H, s, 6‴-CH_3_). ^13^C NMR (150 MHz, DMSO-*d*_6_): δ_C_ 177.4 (C-4), 172.7 (C-1‴), 170.3 (C-5‴), 164.4 (C-7), 161.3 (C-5), 160.1 (C-4′), 156.7 (C-2), 156.5 (C-9), 1133.1 (C-3), 130.9 (C-2′), 120.8 (C-1′), 115.2 (C-3′ and C-5′), 104.0 (C-10), 101.1 (C-1″), 98.9 (C-6), 93.8 (C-8), 76.2 (C-3″), 74.1 (C-2″, 5″), 70.0 (C-4″), 68.9 (C-3‴), 63.2 (C-6″), 45.4 (C-4‴), 45.2 (C-2‴), and 27.3 (C-6‴).

**No. 15** (*p*-coumaric acid): Yellow powder. ^1^H NMR (600 MHz, DMSO-*d*_6_): δ_H_ 7.48–7.51 (3H, m, H-2, 6, 7), 6.79 (2H, d, *J* = 8.5 Hz, H-3 and H-5), and 6.29 (1H, d, *J* = 15.9 Hz, H-8). ^13^C NMR (150 MHz, DMSO-*d*_6_): δ_C_ 168.1 (C-9), 159.7 (C-4), 144.1 (C-7), 130.1 (C-2, 6), 125.3 (C-1), 115.8 (C-3, 5), and 115.6 (C-8).

**No. 16** (ferulic acid): Yellow powder. ^1^H NMR (600 MHz, DMSO-*d*_6_): δ_H_ 7.51 (1H, d, *J* = 15.9 Hz, H-7), 7.27 (1H, d, *J* = 1.8 Hz, H-2), 7.08 (1H, dd, *J* = 8.3, 1.8 Hz, H-6), 6.80 (1H, d, *J* = 8.3 Hz, H-5), 6.37 (1H, d, *J* = 15.9 Hz, H-8), and 3.81 (3H, s, 7-OCH_3_). ^13^C NMR (150 MHz, DMSO-*d*_6_): δ_C_ 168.2 (C-9), 149.3 (C-3), 148.0 (C-4), 144.7 (C-7), 125.9 (C-1), 122.9 (C-6), 115.8 (C-5), 115.7 (C-8), 111.2 (C-2), and 55.8 (7-OCH_3_).

**No. 17** (*cis*-*p*-hydroxyl ethyl cinnamate): Yellow powder. ^1^H NMR (600 MHz, DMSO-*d*_6_): δ_H_ 7.63 (2H, d, *J* = 8.7 Hz, H-2, 6), 6.84 (1H, d, *J* = 12.9 Hz, H-7), 6.75 (2H, d, *J* = 8.7 Hz, H-3, 5), 5.75 (1H, d, *J* = 12.9 Hz, H-8), 4.11 (2H, q, *J* = 7.1 Hz, H-10), and 1.20 (3H, t, *J* = 7.1 Hz, 11-CH_3_). ^13^C NMR (150 MHz, DMSO-*d*_6_): δ_C_ 166.0 (C-9), 158.9 (C-4), 143.0 (C-7), 132.5 (C-2, 6), 125.4 (C-1), 115.5 (C-8), 114.9 (C-3, 5), 59.6 (C-10), and 14.1 (11-CH_3_).

**No. 18** (caffeic acid ethylester): Yellow powder. ^1^H NMR (600 MHz, DMSO-*d*_6_): δ_H_ 7.47 (1H, d, *J* = 15.9 Hz, H-7), 7.05 (1H, d, *J* = 1.8 Hz, H-2), 6.99 (1H, dd, *J* = 8.1, 1.8 Hz, H-6), 6.76 (1H, d, *J* = 8.1 Hz, H-5), 6.25 (1H, d, *J* = 15.9 Hz, H-5), 4.14 (2H, q, *J* = 7.1 Hz, H-10), and 1.23 (3H, t, *J* = 7.1 Hz, 11-CH_3_). ^13^C NMR (150 MHz, DMSO-*d*_6_): δ_C_ 166.6 (C-9), 148.4 (C-3), 145.6 (C-4), 145.0 (C-7), 125.6 (C-4), 121.4 (C-6), 115.8 (C-5), 114.8 (C-2), 114.1 (C-8), 59.7 (C-10), and 14.3 (11-CH_3_).

**No. 19** (*trans*-*p*-hydroxyl ethyl cinnamate): White crystal (chloroform). ^1^H NMR (600 MHz, DMSO-*d*_6_): δ_H_ 9.98 (1h, s, 4-OH), 7.56 (1H, d, *J* = 15.9 Hz, H-7), 7.54 (2H, d, *J* = 8.6 Hz, H-2, 6), 6.79 (2H, d, *J* = 8.6 Hz, H-3, 5), 6.37 (1H, d, *J* = 15.9 Hz, H-8), 4.15 (2H, q, *J* = 7.1 Hz, H-10), and 1.23 (3H, t, *J* = 7.1 Hz, 11-CH_3_). ^13^C NMR (150 MHz, DMSO-*d*_6_): δ_C_ 166.6 (C-9), 159.9 (C-4), 144.6 (C-7), 130.3 (C-2, 6), 125.1 (C-1), 115.8 (C-3, 5), 114.3 (C-8), 59.7 (C-10), and 14.3 (11-CH_3_).

**No. 20** (apigenin-7-O-(6″-(*E*)-p-coumaroyl)-*β*-D-galactopyranoside): Yellow powder. ^1^H NMR (600 MHz, DMSO-*d*_6_): δH 12.97 (1H, s, 5-OH), 7.89 (2H, d, *J* = 8.7 Hz, H-2′, 6′), 7.50 (1H, d, *J* = 15.8 Hz, H-7″), 7.35 (2H, d, *J* = 8.3 Hz, H-2″, 6″), 6.91 (2H, d, *J* = 8.7 Hz, H-3′, 5′), 6.79 (1H, br.s, H-3), 6.77 (1H, d, *J* = 1.8 Hz, H-8), 6.68 (2H, d, *J* = 8.3 Hz, H-3″, 5″), 6.47 (1H, d, *J* = 1.8 Hz, H-6), 6.33 (1H, d, *J* = 15.8 Hz, H-8″), 5.19 (1H, d, *J* = 7.3 Hz, H-1‴),4.51 (1H, d, *J* = 11.3 Hz, H-6‴), 4.21 (1H, m, H-6‴), 3.86 (1H, t, *J* = 8.1 Hz, H-5‴), 3.43 (1H, m, H-3‴), 3.39 (1H, m, H-2‴), and 3.32 (1H, m, H-4‴). ^13^C NMR (150 MHz, DMSO-*d*_6_): δ_C_ 182.0 (C-4), 166.6 (C-9″), 164.3 (C-2), 162.8 (C-7), 161.5 (C-5), 161.3 (C-4′), 159.9 (C-4″), 157.0 (C-9), 145.0 (C-7″), 130.2 (C-2″, 6″), 128.6 (C-2′, 6′), 125.0 (C-1″), 121.1 (C-1′), 116.1 (C-3′, 5′), 115.8 (C-3″, 5″), 113.8 (C-8″), 105.5 (C-10), 99.6 (C-6, 1‴), 94.8 (C-8), 76.5 (C-5‴), 74.0 (C-3‴), 73.1 (C-2‴), 70.0 (C-4‴), and 63.6 (C-6‴).

**No. 21** (apigenin-7-O-(6″-*E*-p-coumaroyl)-*β*-D-glucopyranoside): Yellow crystal (methanol). ^1^H NMR (600 MHz, DMSO-*d*_6_): δH 12.97 (1H, s, 5-OH), 10.15 (2H, s, 4′, 4″-OH), 7.93 (2H, d, *J* = 8.7 Hz, H-2′, 6′), 7.50 (1H, d, *J* = 15.9 Hz, H-7″), 7.36 (2H, d, *J* = 8.6 Hz, H-2″, 6″), 6.92 (2H, d, *J* = 8.7 Hz, H-3′, 5′), 6.80–6.81 (2H, m, H-3, 8), 6.67 (2H, d, *J* = 8.6 Hz, H-3″, 5″), 6.48 (1H, d, *J* = 2.0 Hz, H-6), 6.33 (1H, d, *J* = 15.9 Hz, H-8″), 5.53 (1H, br.s, 4‴-OH), 5.40 (1H, br.s, 3‴-OH), 5.30 (1H, br.s, 2‴-OH), 5.17 (1H, d, *J* = 7.5 Hz, H-1‴), 4.48 (1H, d, *J* = 10.4 Hz, H-6‴), 4.18 (1H, dd, *J* = 10.4, 7.2 Hz, H-6‴), 3.84 (1H, m, H-5‴), 3.34–3.39 (2H, m, H-2‴, 3‴), and 3.28 (1H, m, H-4‴). ^13^C NMR (150 MHz, DMSO-*d*_6_): δ_C_ 182.0 (C-4), 166.5 (C-9″), 164.3 (C-2), 162.7 (C-7), 161.4 (C-4′), 161.2 (C-5), 159.8 (C-4″), 156.9 (C-9), 145.0 (C-7″), 130.1 (C-2″, 6″), 128.5 (C-2′, 6′), 124.9 (C-1″), 121.0 (C-1′), 116.0 (C-3′, 5′), 115.7 (C-3″, 5″), 113.8 (C-8″), 105.4 (C-10), 103.1 (C-3), 99.5 (C-6, 1‴), 94.7 (C-8), 76.3 (C-5‴), 73.9 (C-3‴), 73.0 (C-2‴), 70.0 (C-4‴), and 63.5 (C-6‴).

**No. 22** (apigenin-7-O-(3″,6″-di-(*E*)-p-coumaroyl)-*β*-D-galactopyranoside): Yellow crystal (methanol). ^1^H NMR (600 MHz, DMSO-*d*_6_): δ_H_ 13.01 (1H, s, 5-OH), 7.95 (2H, d, *J* = 8.8 Hz, H-2′, 6′), 7.61 (1H, d, *J* = 15.9 Hz, H-7″), 7.58 (2H, d, *J* = 8.6 Hz, H-2″, 6″), 7.51 (1H, d, *J* = 15.9 Hz, H-7″), 7.40 (2H, d, *J* = 8.6 Hz, H-2‴, 6‴), 6.93 (2H, d, *J* = 8.8 Hz, H-3′, 5′), 6.85 (1H, d, *J* = 1.8 Hz, H-8), 6.84 (1H, s, H-3), 6.81 (2H, d, *J* = 8.6 Hz, H-3″, 5″), 6.69 (2H, d, *J* = 8.6 Hz, H-3‴, 5‴), 6.52 (1H, d, *J* = 1.8 Hz, H-8), 6.46 (1H, d, *J* = 15.9 Hz, H-8″), 6.36 (1H, d, *J* = 15.9 Hz, H-8″), 5.78 (1H, d, *J* = 5.0 Hz, H-1″″), 5.64 (1H, d, *J* = 5.7 Hz, H-3″″), 5.38 (1H, d, *J* = 7.7 Hz,, 4‴-OH), 5.12 (1H, t, *J* = 9.3 Hz, 2‴-OH), 4.47 (1H, d, *J* = 11.8 Hz, H-6″″), 4.23 (1H, dd, *J* = 11.8, 6.7 Hz, H-6″″), 4.03 (1H, m, H-5″″), and 3.52–3.60 (2H, m, H-2″″, 4″″). ^13^C NMR (150 MHz, DMSO-*d*_6_): δ_C_ 182.0 (C-4), 166.4 (C-9″), 166.1 (C-9‴), 164.3 (C-2), 162.4 (C-7), 161.4 (C-4′), 161.2 (C-5), 159.8 (C-4″, 4‴), 156.9 (C-9), 145.0 (C-7‴), 144.7 (C-7″), 114.7 (C-8″), 130.3 (C-2″, 6″), 130.2 (C-2‴, 6‴), 128.6 (C-2′, 6′), 125.2 (C-1″), 124.9 (C-1‴), 121.0 (C-1′), 116.0 (C-3′, 5′), 115.8 (C-3″, 5″), 115.7 (C-3‴, 5‴), 113.7 (C-8‴), 105.5 (C-10), 103.1 (C-3), 99.5 (C-1″″), 99.1 (C-6), 94.8 (C-8), 76.9 (C-3″″), 73.6 (C-5″″), 71.1 (C-2″″), 68.0 (C-4″″), and 63.4 (C-6″″).

**No. 23** (apigenin-7-O-(3″-*p*-coumaryl)-glucoside): Yellow powder. ^1^H NMR (600 MHz, DMSO-*d*_6_): δ_H_ 12.99 (1H, s, 5-OH), 10.41 (1H, s, 4′-OH), 10.01 (1H, s, 4″-OH), 7.97 (2H, d, *J* = 8.8 Hz, H-2′, 6′), 7.59 (1H, d, *J* = 15.9 Hz, H-7″), 7.57 (2H, d, *J* = 8.5 Hz, H-2″, 6″), 6.95 (2H, d, *J* = 8.8 Hz, H-3′, 5′), 6.88 (1H, s, H-3), 6.87 (1H, d, *J* = 1.9 Hz, H-8), 6.81 (2H, d, *J* = 8.5 Hz, H-3″, 5″), 6.48 (1H, d, *J* = 1.9 Hz, H-6), 6.44 (1H, d, *J* = 15.9 Hz, H-8″), 5.28 (1H, d, *J* = 7.7 Hz, H-1‴), 5.07 (1H, t, *J* = 9.4 Hz, H-3‴), 3.72 (1H, m, H-5‴), 3.63 (1H, m, H-4‴), 3.54 (1H, m, H-6‴), 3.51 (1H, m, H-6‴), 3.48 (1H, m, H-2‴). ^13^C NMR (150 MHz, DMSO-*d*_6_): δ_C_ 182.0 (C-4), 166.2 (C-9″), 164.3 (C-2), 162.7 (C-7), 161.4 (C-5), 161.2 (C-4′), 159.7 (C-4″), 156.9 (C-9), 144.5 (C-7″), 130.2 (C-2″, 6″), 128.6 (C-2′, 6′), 125.2 (C-1″), 121.0 (C-1′), 116.0 (C-3′, 5′), 115.8 (C-3″, 5″), 114.8 (C-8″), 105.5 (C-10), 103.2 (C-3), 99.5 (C-6, 1‴), 94.9 (C-8), 77.2 (C-3‴), 76.9 (C-5‴), 71.2 (C-2‴), 67.5 (C-4‴), and 60.2 (C-6‴).

**No. 24** (palhinoside A): Yellow powder. ^1^H NMR (600 MHz, DMSO-*d*_6_): δ_H_ 12.80 (2H, s, 5-OH), 7.93 (2H, d, *J* = 8.6 Hz, H-2′, 6′), 7.73 (2H, d, *J* = 8.6 Hz, H-2′, 6′), 6.92 (2H, d, *J* = 8.7 Hz, H-3′, 5′), 6.83 (2H, d, *J* = 8.7 Hz, H-3′, 5′), 6.75/6.43 (1H, s, H-8), 6.66/6.64 (1H, s, H-3), 6.37/6.18 (1H, s, H-6), 6.35 (4H, m, H-2b/6b, 3b/5b), 6.32 (2H, d, *J* = 7.8 Hz, H-2a/6a), 6.25 (2H, d, *J* = 8.4 Hz, H-3a/5a), 5.35/5.03 (1H, d, *J* = 7.5Hz, H-1″), 4.68/4.32/4.04/3.29 (1H, d, *J* = 10.9/11.8/9.2 Hz, H-6″), 3.92/3.76 (1H, dd, *J* = 11.0, 8.3 Hz, H-5″), 3.46~3.43 (2H, m, H-3″), 3.34 (2H, d, *J* = 8.5 Hz, H-2″), 3.28/3.17 (1H, m, H-4″), 4.02 (2H, d, *J* = 9.5 Hz, H-7b), 3.86 (1H, dd, *J* = 10.0, 5.4 Hz, H-7a), 3.60 (1H, t, *J* = 9.5 Hz, H-8b), and 3.37 (1H, s, H-8a). ^13^C NMR (150 MHz, DMSO-*d*_6_): δ_C_ 181.7/181.6 (C-4), 172.5 (C-9b), 172.0 (C-9a), 162.7/162.1 (C-7), 164.2/164.1 (C-2), 161.9/161.6 (C-4′), 161.2/160.8 (C-5), 156.7/156.5 (C-9), 155.3 (C-4a, 4b), 130.1 (C-1a), 128.7 (C-1b), 128.6 (C-2a, 2b, 6a, 6b), 128.4 (C-2′, 6′), 120.9/120.8 (C-1′), 116.2/116.0 (C-3′, 5′), 114.6 (C-3a, 3b, 5a, 5b), 105.1 (C-10), 102.9/102.8 (C-3), 99.4/99.2 (C-6), 99.1/98.9 (C-1″), 94.2/94.0 (C-8), 76.6/75.9 (C-3″), 73.8/73.7 (C-5″), 72.9 (C-2″), 70.5/70.4 (C-4″), 65.1/64.5 (C-6″), 43.4 (C-7a), 43.1 (C-7b), 43.0 (C-8a), and 42.2 (C-8b).

**No. 25** (tiliroside): Yellow powder. ^1^H NMR (600 MHz, DMSO-*d*_6_): δ_H_ 12.58 (1H, s, 5-OH), 10.27 (2H, s, 4′, 4″-OH), 8.01 (2H, d, *J* = 8.7 Hz, H-2′, 6′), 7.35–7.37 (3H, m, H-2″, 6″, 7″), 6.87 (2H, d, *J* = 8.7 Hz, H-3′, 5′), 6.80 (2H, d, *J* = 8.4 Hz, H-3″, 5″), 6.12 (1H, d, *J* = 15.9 Hz, H-8″), 6.39 (1H, d, *J* = 1.6 Hz, H-8), 6.16 (1H, d, *J* = 1.6 Hz, H-6), 5.48 (1H, d, *J* = 7.4 Hz, H-1‴), 4.31 (1H, d, *J* = 11.5 Hz, H-6‴), 4.08 (1H, m, H-6‴), 3.44 (1H, m, H-2‴), and 3.22–3.32 (3H, m, H-3‴, 4‴, 5‴). ^13^C NMR (150 MHz, DMSO-*d*_6_): δ_C_ 177.5 (C-4), 166.3 (C-9″), 164.4 (C-7), 161.3 (C-5), 160.1 (C-4′), 159.9 (C-4″), 156.7 (C-2), 156.5 (C-9), 144.8 (C-7″), 133.2 (C-3), 131.0 (C-2″, 6″), 130.3 (C-2′, 6′), 125.1 (C-1″), 120.9 (C-1′), 115.9 (C-3′, 5′), 115.3 (C-3″, 5″), 113.8 (C-8″), 104.0 (C-1‴), 101.2 (C-10), 99.0 (C-6), 93.9 (C-8), 76.4 (C-3‴), 74.4 (C-2‴), 74.3 (C-5‴), 70.1 (C-4‴), and 63.2 (C-6‴).

**No. 26** (*cis*-tiliroside): Yellow powder. ^1^H NMR (600 MHz, DMSO-*d*_6_): δ_H_ 12.52 (1H, s, 5-OH), 7.95 (2H, d, *J* = 8.6 Hz, H-2′, 6′), 7.55 (2H, d, *J* = 8.5 Hz, H-2″, 6″), 6.85 (2H, d, *J* = 8.6 Hz, H-3′, 5′), 6.70 (2H, d, *J* = 8.5 Hz, H-3″, 5″), 6.67 (1H, d, *J* = 12.9 Hz, H-7″), 6.35 (1H, br.s, H-8), 6.19 (1H, br.s, H-6), 5.47 (1H, d, *J* = 12.9 Hz, H-8″), 5.41 (1H, d, *J* = 7.5 Hz, H-1‴), 4.17 (1H, d, *J* = 10.2 Hz, H-6‴), 4.08 (1H, m, H-6‴), 3.37 (1H, m, H-2‴), and 3.16–3.25 (3H, m, H-3‴, 4‴, 5‴). ^13^C NMR (150 MHz, DMSO-*d*_6_): δ_C_ 177.3 (C-4), 165.4 (C-9″), 164.9 (C-7), 161.2 (C-5), 160.1 (C-4′), 158.9 (C-4″), 156.5 (C-2, 9), 143.8 (C-7″), 133.0 (C-3), 132.7 (C-2″, 6″), 130.8 (C-2′, 6′), 125.3 (C-1″), 120.8 (C-1′), 115.1 (C-3′, 5′), 114.8 (C-3″, 5″), 114.6(C-8″), 103.7 (C-10), 101.1 (C-1‴), 98.9 (C-6), 93.8 (C-8), 76.3 (C-3‴), 74.1 (C-2‴), 74.0 (C-5‴), 70.0 (C-4‴), and 62.7 (C-6‴).

**No. 27** (anisofolin A): Yellow powder. ^1^H NMR (600 MHz, DMSO-*d*_6_): δ_H_ 12.96 (1H, s, 5-OH), 7.93 (2H, d, *J* = 8.6 Hz, H-2′, 6′), 7.59 (1H, d, *J* = 16.0 Hz, H-7″), 7.56 (2H, d, *J* = 8.6 Hz, H-2″, 6″), 7.49 (1H, d, *J* = 15.8 Hz, H-7″), 7.36 (2H, d, *J* = 8.6 Hz, H-2‴, 6‴), 6.92 (2H, d, *J* = 8.6 Hz, H-3′, 5′), 6.84 (1H, d, *J* = 2.9 Hz, H-8), 6.82 (1H, s, H-3), 6.80 (2H, d, *J* = 8.6 Hz, H-3″, 5″), 6.67 (2H, d, *J* = 8.6 Hz, H-3‴, 5‴), 6.49 (1H, d, *J* = 2.9 Hz, H-8), 6.43 (1H, d, *J* = 16.0 Hz, H-8″), 6.33 (1H, d, *J* = 15.8 Hz, H-8″), 5.35 (1H, d, *J* = 7.7 Hz, H-1″″), 5.11 (1H, t, *J* = 9.4 Hz, H-3″″), 4.46 (1H, d, *J* = 11.0 Hz, H-6″″), 4.21 (1H, m, H-6″″), and 3.36–4.01 (3H, m, H-2″″, 4″″, 5″″). ^13^C NMR (150 MHz, DMSO-*d*_6_): δ_C_ 182.2 (C-4), 166.7 (C-9′′), 166.5 (C-9‴), 164.6 (C-2), 162.6 (C-7), 161.7 (C-5), 161.4 (C-4′), 160.1 (C-4‴),160.0 (C-4′′), 157.1 (C-9), 145.3 (C-7‴), 145.0 (C-7′′), 130.5 (C-2′′, 6′′), 130.4 (C-2‴, 6‴), 128.8 (C-2′, 6′), 125.4 (C-1′′), 125.1 (C-1‴), 121.1 (C-1′), 116.3 (C-3′, 5′), 116.1 (C-3′′, 5′′), 116.0 (C-3‴, 5‴), 114.9 (C-8′′), 113.8 (C-8‴), 105.7 (C-10), 103.2 (C-3), 99.8 (C-6), 99.3 (C-1″″), 95.0 (C-8), 77.1 (C-5″″), 73.8 (C-3″″), 71.3 (C-2″″), 68.3 (C-4″″), and 63.3 (C-6″″). 

**No. 28** (vanillic acid): Colorless crystal (methanol). ^1^H NMR (600 MHz, DMSO-*d*_6_): δ_H_ 12.48 (1H, s, 1-COOH), 9.83 (1H, s, 4-OH), 7.43–7.46 (2H, m, H-2, 6), 6.84 (1H, d, *J* = 8.1 Hz, H-5), and 3.80 (3H, s, 3-OCH_3_). ^13^C NMR (150 MHz, DMSO-*d*_6_): δ_C_ 167.3 (C-7), 151.1 (C-3), 147.3 (C-4), 123.5 (C-6), 121.7 (C-1), 115.1 (C-2), 112.7 (C-5), and 55.6 (3-OCH_3_).

**No. 29** (syringic acid): Colorless crystal (methanol). 1H NMR (600 MHz, DMSO-*d*_6_): δH 7.21 (2H, s, H-2, 6) and 3.80 (6H, s, 3, 5-OCH3). 13C NMR (150 MHz, DMSO-*d*_6_): δ_C_ 167.3 (C-7), 147.4 (C-4), 140.1 (C-3, 5), 120.6 (C-1), 106.9 (C-2, 6), and 56.0 (3, 5-OCH3).

**No. 30** (4-(9H-B-carbolin-1-yl)-4-oxobut-2-enoic acid methyl ester): Yellow powder: ^1^H NMR (600 MHz, DMSO-*d*_6_): δ_H_ 12.17 (1H, s, NH), 8.69 (1H, d, *J* = 4.9 Hz, H-3), 8.60 (1H, d, *J* = 15.9 Hz, H-11), 8.53 (1H, d, *J* = 4.8 Hz, H-4), 8.34 (1H, d, *J* = 7.8 Hz, H-5), 7.83 (1H, d, *J* = 8.2 Hz, H-8), 7.62 (1H, m), 7.34 (1H, m), 6.94 (1H, d, *J* = 15.9 Hz, H-12), and 3.83 (3H, s, OCH_3_). ^13^C NMR (150 MHz, DMSO-*d*_6_): δ_C_ 189.2 (C-10), 165.6 (C-13), 141.9 (C-8a), 137.9 (C-3), 136.1 (C-11), 135.2 (C-9a), 135.1 (C-1), 131.3 (C-4a), 130.0 (C-12), 129.2 (C-7), 122.0 (C-5), 120.6 (C-6), 120.4 (C-4), 119.9 (C-4b), 113.2 (C-8), and 52.3 (13-OCH_3_).

The information of 30 phytochemicals from the LSD fraction are exhibited in the Supplementary Materials (Table S3). Among them, Nos. **1**–**3**, **4**–**14**, **15**–**19**, **20**–**27**, and **28**–**30** belong to the phenylethanol glycosides, flavonoids, phenylpropanoids, flavone-phenylpropanoids, and others, respectively. To the best of our knowledge, this is the first report of Nos. **3**, **13**, **17**, **19**, **24**, **26**, and **30** in the *Lamiaceae* family. Moreover, Nos. **1**, **4**–**9**, **11**, **12**, **15**, **18**, **21**, **23**, and **27**–**29** were reported for the first time from the *Lagopsis* genus.

### 2.3. Anti-MI Activity of the Compounds

**Figure 4:**
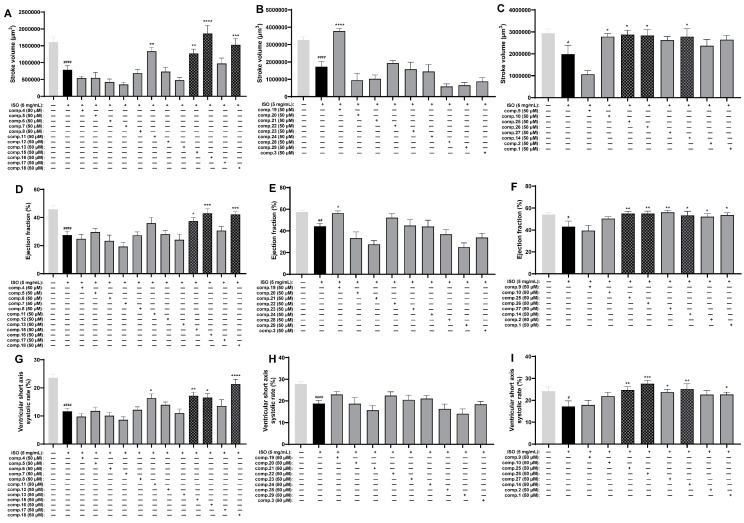
Results of the anti-MI activity evaluation of Comp. **1**–**30** in zebrafish (*n* = 6–11). Effects of Comp. **1**–**30** on stroke volume (**A**–**C**), ejection fraction (**D**–**F**), and ventricular short-axis systolic rate (**G**–**I**). The data are expressed as the means ± SEMs. ^#^ *p* < 0.05, ^##^ *p* < 0.01, and ^####^ *p* < 0.0001 vs. the C group. * *p* < 0.05, ** *p* < 0.01, *** *p* < 0.001, and **** *p* < 0.0001 vs. the ISO group.

The impact of 30 substances on myocardial infarction injury was assessed using the ISO-induced zebrafish MI model. As shown in Figure 4, compared with those of the control group, the three indices (stroke volume, ejection fraction, and ventricular short-axis systolic) of the ISO group were markedly lower. In contrast to the ISO-treated group, Nos. **9**, **10**, **11**, **14**–**16**, **18**, **19**, **25**, and **26** had apparent reparative effects on stroke volume (Figure 4A–C); Nos. **1**, **2**, **14**–**16**, **18**, **19**, and **25**–**27** had marked effects on the ejection fraction (Figure 4D–F); and Nos. **1**, **11**, **14**–**16**, **18**, and **25**–**27** had apparent effects on the ventricular short-axis systolic rate (Figure 4G–I). In combination with the above results, six phytochemicals, **14**–**16**, **18**, **25**, and **26**, significantly improved all three indices in the ISO-induced zebrafish model.

**Figure 5:**
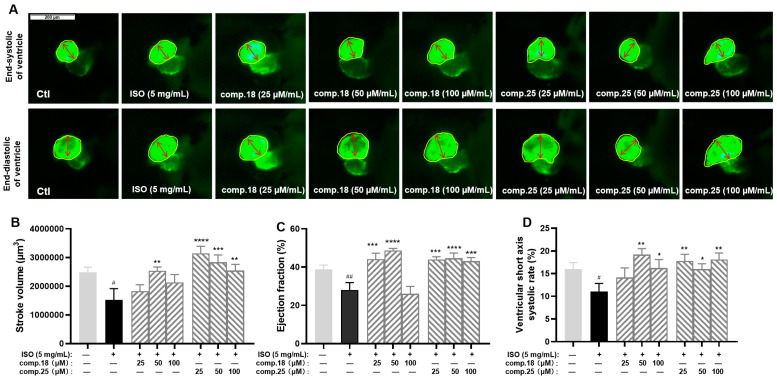
Results of anti-MI activity evaluation for tiliroside and caffeic acid ethyl ester in zebrafish (*n* = 6–10). Representative images of the end-systolic and end-diastolic regions of zebrafish ventricles in each group ((**A**), yellow represents the ventricular area and red represents the ventricular short axis length). Effects of tiliroside and caffeic acid ethyl ester on stroke volume (**B**), ejection fraction (**C**), and ventricular short-axis systolic rate (**D**). The data are expressed as the means ± SEMs. ^#^ *p* < 0.05 and ^##^ *p* < 0.01 vs. the C group. * *p* < 0.05, ** *p* < 0.01, *** *p* < 0.001, and **** *p* < 0.0001 vs. the ISO group.

On the basis of the therapeutic effects and yields of these compounds, caffeic acid ethyl ester (No. **18**) and tiliroside (No. **25**), representing lignan and flavonoid glycosides, respectively, were selected for further activity comparisons. As shown in Figure 5, tiliroside (No. **25**) had significant therapeutic effects on the stroke volume, ejection fraction, and the ventricular short-axis systolic rate at concentrations ranging from 25 to 100 µM (Figure 5B–D). Comparatively speaking, the anti-MI activity of caffeic acid ethyl ester (No. **18**) was weaker than that of tiliroside. Caffeic acid ethyl ester (No. **18**) at a concentration of 50 µM had an obvious effect on all three indices. The indices of the 100 µM caffeic acid ethyl ester-treated group showed varying degrees of decline compared with those of the 50 µM caffeic acid group, especially the ejection fraction (Figure 5B–D). In addition, at a concentration of 100 µM, when treated with caffeic acid ethyl ester (No. **18**), zebrafish exhibited abnormal behavior. Thus, caffeic acid ethyl ester (No. **18**) may be toxic when it is used at this concentration in zebrafish.

### 2.4. Molecular Docking Experiment of Tiliroside

**Figure 6:**
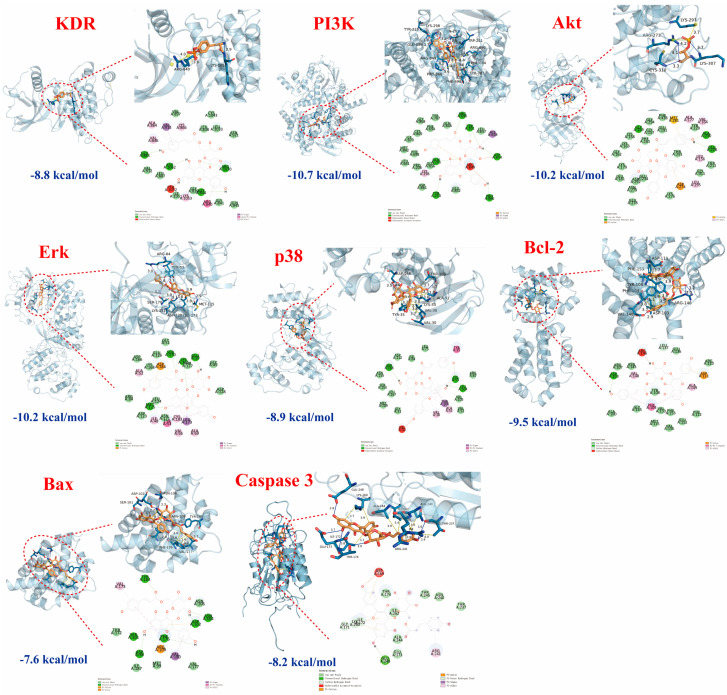
Molecular docking results of tiliroside (blue lines represent hydrogen bonds, yellow lines represent hydrophobic interactions, and yellow lines with spheres indicate π-cation interactions). The binding affinity of tiliroside (**25**) with the proteins KDR, PI3K, Akt, Erk, p38, Bcl-2, Bax, and Caspase3.

We further employed a molecular docking technique to verify the binding affinity of tiliroside (Number **25**) with the proteins KDR, PI3K, Akt, Erk, p38, Bcl-2, Bax, and Caspase3, in light of the essential roles of the PI3K-Akt and MAPK pathways, as well as apoptosis, associated with MI. A binding energy lower than −5.0 kcal/mol indicated strong interactions [21]. As shown in Figure 6, No. **25** had a strong binding ability with these nine proteins, and their binding energies were −8.8, −10.7, −10.2, −10.2, −8.9, −9.5, −7.6, and −8.2 kcal/mol, respectively, which were lower than −7.0 kcal/mol. These findings implied that the anti-MI activity of No. **25** may be an essential active constituent in the LSD fraction of *L. supina*, potentially involved in regulating the PI3K-Akt and MAPK signaling pathways.

### 2.5. RT-qPCR Validation of Tiliroside

**Figure 7:**
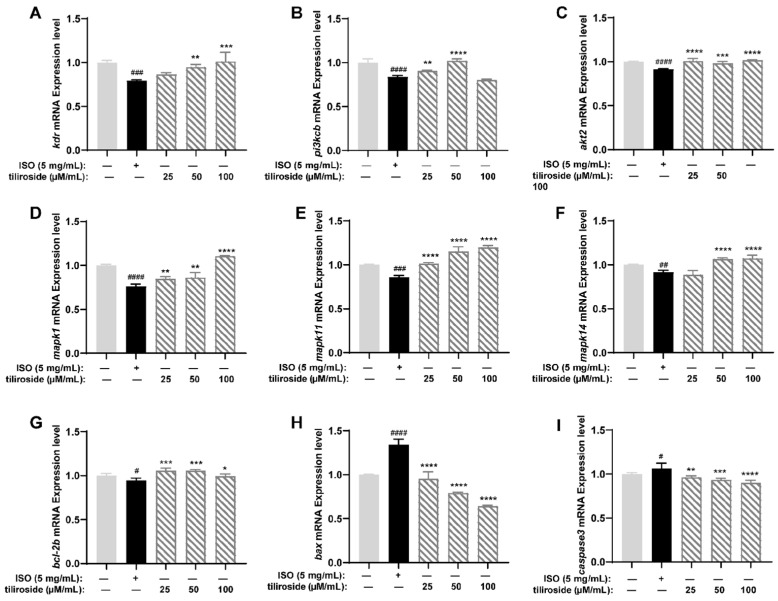
Results of RT-qPCR for tiliroside (*n* = 3). Gene expression levels of *kdr* (**A**), *pik3cb* (**B**), *akt2* (**C**), *mapk1* (**D**), *mapk11* (**E**), *mapk14* (**F**), *bcl-2b* (**G**), *bax* (**H**), and *caspase3* (**I**). The data are expressed as the means ± SDs. ^#^ *p* < 0.05, ^##^ *p* < 0.01, ^###^ *p* < 0.001 and ^####^ *p* < 0.0001 vs. the C group. * *p* < 0.05, ** *p* < 0.01, *** *p* < 0.001 and **** *p* < 0.0001 vs. the ISO group.

Through the results of activity evaluation and molecular docking experiments, we selected nine related genes, namely, *kdr*, *pik3cb*, *akt2*, *mapk1*, *mapk11*, *mapk14*, *bcl-2b*, *bax*, and *caspase3*, for PCR analysis to reveal the mechanism of action of tiliroside (No. **25**). As shown in Figure 7, compared with the control, ISO significantly inhibited the expression of *kdr*, which further inhibited the PI3K-Akt and MAPK signaling pathways. ISO also reduced the expression of the *pik3cb* and *akt2* genes in the PI3K-Akt pathway and the expression of the *mapk1*, *mapk11,* and *mapk14* genes in the MAPK pathway. With respect to apoptosis factors, ISO increased the expression of *bax* and *caspase3* and decreased the expression of *bcl-2b*. On the basis of these experimental results, we speculated that ISO inhibits the gene expression of the PI3K-Akt and MAPK pathways, increases the expression levels of the proapoptotic factors *bax* and *caspase3*, and decreases the expression level of the anti-apoptotic factor *bcl-2b*. Treatment with tiliroside (**25**) activated the expression of genes such as *kdr*, *pik3cb*, *akt2*, *mapk1*, *mapk11*, *mapk14*, and *bcl-2b* and inhibited the expression of *bax* and *caspase3*, indicating that it could activate the PI3K-Akt and MAPK pathways and inhibit cell apoptosis.

## 3. Discussion

Studying the chemical diversity and multifunctional biological activity of TCMs has always been an important area of Chinese medicine research [49]. Numerous clinical procedures have demonstrated the clear therapeutic benefits and clinical usefulness of TCM in treating complex illnesses, including cancers, digestive disorders, cardiovascular and cerebrovascular disorders, and more [50]. TCM *L. supina* can improve blood circulation in patients′ bodies and nourish the blood, and its extract has been proven to contain multiple active medicinal ingredients. Our previous work revealed that LSD has a significant therapeutic effect on MI, but more research is needed on the active substances involved and corresponding underlying mechanism [20]. In this study, for the first time, we systematically delved into the pharmacodynamics material basis of the anti-MI effects of LSD and its potential mechanisms of action. The present work presented four significant innovations: (1) Thirty phytochemicals were isolated and identified from the anti-MI active fraction (LSD) of *L. supina*. (2) This study is the first to systematically elucidate the substance basis by which *L. supina* protects against MI. (3) This is the first study of the anti-MI activity of tiliroside. (4) Tiliroside showed the strongest anti-MI activity by activating the *kdr*-mediated PI3K-Akt and MAPK signaling pathways in ISO-induced zebrafish.

At present, experimental models for studying MI include in vitro cell models (H9C2 cells, hUC-MSCs, etc.) and in vivo animal models, such as zebrafish and rats [15,51,52,53]. Cell models fail to precisely replicate and mimic the complex environment and processes seen in living organisms, including drug absorption, distribution, metabolism, and excretion [54]. The long experimental period and the need for extensive sample quantities limited the application of the rat model in high-throughput drug screening, especially for natural products with small amounts [55]. The transparency of zebrafish allows intuitive observation of heart development and drug effects, and the use of a genetically modified zebrafish strain labeled with fluorescent markers further enhances the visualization of heart morphology [55]. In addition, during early development, it can be passively diffused and oxygenated, allowing researchers to better understand the mechanisms of heart development and function and explore changes in cardiac phenotype diseases [11]. This study induced an MI model in zebrafish via ISO to evaluate therapeutic effects on the basis of three indices: stroke volume, ejection fraction, and ventricular short-axis systolic rate [15,56]. Through activity evaluation, we confirmed that LSD is the main active fraction of *L. supina* and can significantly improve the stroke volume and ejection fraction at a concentration of 100 µg/mL. A chemical investigation of LSD resulted in the isolation of 30 phytochemicals (Nos. **1**–**30**), including phenylethanoid glycosides (Nos. **1**–**3**), flavonoid glycosides (Nos. **4**–**14** and **20**–**27**), lignans (Nos. **15**–**19**), glycosidebenzoic acids (Nos. **28–29**), and alkaloids (No. **30**). Among them, Nos. **3**, **13, 17**, **19**, **24**, **26**, and **30** were first isolated from the *Lamiaceae* family and Nos. **1**, **4–9**, **11**, **12**, **15**, **18**, **21**, **23**, and **27**–**29** were isolated for the first time from the *Lagopsis* genus. The results from the zebrafish MI model revealed six bioactive compounds, three lignans (*p*-coumaric acid (No. **15**), ferulic acid (No. **16**), and caffeic acid ethyl ester (No. **18**)), and three flavonoid glycosides (kaempferol-3-O-*β*-D-glucopyranoside-6″-(3-hydroxy-3-methylglutarate) (No. **14**), tiliroside (No. **25**), and *cis*-tiliroside (No. **26**)). As a result, caffeic acid ethyl ester (**18**) and tiliroside (**25**) were selected for further evaluation for anti-MI at a gradient of dose concentrations with good therapeutic effects and sufficient yields, representing two types of structures. The experimental results suggested that tiliroside (No. **25**) had a better anti-MI effect than did caffeic acid ethyl ester (No. **18**), and this is also the first discovery of anti-MI activity for tiliroside.

MI can be treated by targeting PI3K-Akt, MAPK, Integrin/FAK, and other signaling pathways [5]. PI3K-Akt is one of the important pathways affecting the onset and progression of metabolic cardiovascular diseases [57]. Activating this whole pathway can result in anti-apoptotic effects on myocardial cells. The MAPK pathway controls numerous important physiological and pathological processes, including the proliferation, growth, and differentiation of cardiac resident cells, with apoptosis being one of the affected aspects [57]. Erk and p38, the main families of MAPKs, have been extensively studied for their ability to regulate cardiomyocyte apoptosis [5]. The activation of Erk could affect cell growth and survival to exert a protective effect on cells and mediate the anti-apoptotic function of various molecules, such as the *α*1-adrenergic receptor [5]. Previous studies have confirmed that activating the p38 MAPK signaling pathway can restore metabolic imbalances in myocardial cells [58,59,60]. In addition to triggering biological reactions such as endothelial cell migration, mitosis, and survival, KDR activation can control several signaling pathways, including the PI3K-Akt and MAPK pathways [10,61]. Therefore, finding drugs that regulate the *kdr*-mediated PI3K-Akt and MAPK pathways may be important for exploring drugs for treating MI.

Our previous work also proved that LSD could reduce the damage of MI through PI3K-Akt and MAPK. In this study, we focused on genes involved in the *kdr*-mediated PI3K-Akt and MAPK pathways to explore the mechanism by which tiliroside (No. **25**) has anti-MI activity. KDR is a receptor protein on the cell membrane that can induce downstream PI3K [62]. The activation of *pik3cb* can upregulate the expression of the *akt* gene, which helps maintain energy production in hypoxic–ischemic hearts and inhibits myocardial cell apoptosis [5]. PI3K-Akt signaling pathway activation can inhibit the binding of BAD to Bcl-2, interfere with the proapoptotic effect of BAD, and significantly inhibit the production of cell apoptosis by phosphorylating and inactivating caspase9 [5,63]. KDR also regulates the MAPK signaling pathway and is one of its downstream pathways [64]. In addition, the activation of Erk can reduce bax content in cells, increase bcl-2 levels, and protect the heart from cell apoptosis [65]. In summary, the *kdr*-mediated PI3K-Akt and MAPK signaling pathways can affect the expression of genes related to cell apoptosis [5,57]. Tiliroside has a strong binding ability with the KDR, PI3K, Akt, Erk, p38, Bcl-2, Bax, and Caspase3 proteins, all of which have binding energies lower than −7.0 kcal/mol. RT-qPCR was further used to validate the effects of tiliroside on nine related genes. Consequently, tiliroside markedly upregulated the expression levels of the *kdr*, *pik3cb*, *akt2*, *mapk1*, *mapk11*, *mapk14*, and *bcl-2b* genes and strongly downregulated the expression levels of the *bax* and *caspase3* genes. These results suggested that tiliroside exhibited an anti-MI effect by activating the *kdr*-mediated PI3K-Akt and MAPK signaling pathways, confirming the above molecular docking analysis results.

Tiliroside (**25**) belongs to glycoside flavonoids and can be transported to cells through biological transport [66]. Research has shown that flavonoid compounds have rich biological activities, including antioxidant, immune regulatory, anticancer, and antiparasitic activities [66]. It has been discovered that various flavonoids can treat myocardial ischemia, such as proanthocyanidins, naringenin, quercetin, morin, genistein, theaflavin, baicalein, and luteolin [67]. Tiliroside was found in plants, and its activity and mechanism have been reported in many papers so far, including anti-inflammatory, hepatoprotective, antioxidant, antiobesity, anti-diabetes, etc. [68]. This is the first discovery of its anti-MI effect. Previous research discovered that heptaacetylotiliroside has antiproliferative activity, tiliroside′s derivatives with a modified phenolic ring in the cinnamoyl group have antidiabetic activity, and 7c-(E)-(6-(5,7-dihydroxy-2-(4-hydroxyphenyl)-4-oxo-4H-chromen-3-yloxy)-3,4,5-trihydroxytetra-hydro2H-pyran-2-yl)methyl 3-(4-cyano phenyl)acrylate and 3-O-[(E)-4-(4-cyanophenyl)-2-oxobut-3-en-1-yl]-kaempferol could have antidiabetic and antiobesity effects [68]. At present, there is no research on the anti-MI activity for tiliroside derivatives. Our research provides a new possibility for further developing tiliroside and its derivatives to treat MI.

**Figure 8:**
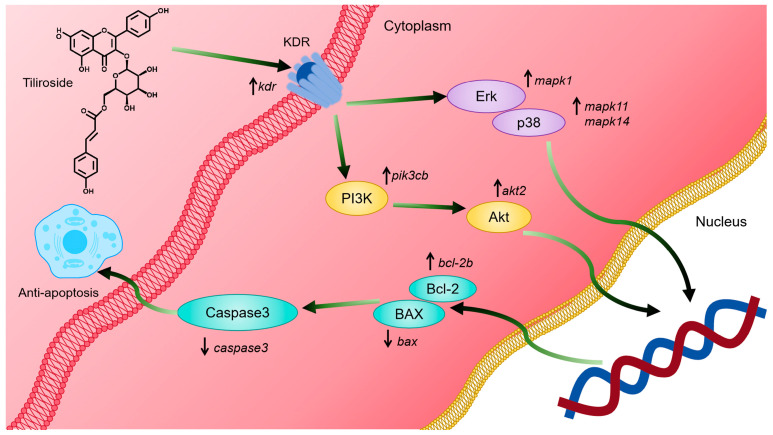
Mechanistic diagram of the alleviation of myocardial ischemia by tiliroside. Tiliroside (**25**) can activate the *kdr*-mediated PI3K-Akt and MAPK signaling pathways, thereby inhibiting cell apoptosis and alleviating ISO-induced MI.

These results indicate that tiliroside (**25**) can activate the *kdr*-mediated PI3K-Akt and MAPK signaling pathways, thereby inhibiting cell apoptosis and alleviating ISO-induced MI in zebrafish (Figure 8). However, this study has several limitations: (1) Western blot and gene-editing experiments need to be performed to confirm the binding targets of tiliroside (**25**). (2) An experiment in a rat model can be conducted to verify the efficacy and mechanism of tiliroside (**25**) in treating MI. (3) Other active numbers with small amounts, such as **18**, can be enriched to further develop their application value in treating MI.

## 4. Materials and Methods

### 4.1. Materials and Reagents

Analytical grade methanol, ethanol, methylene chloride, petroleum ether, and ethyl acetate were obtained from Xilong Chemical Co., Ltd. (Guangxi, China). Isoprenaline hydrochloride was acquired from Beijing Solarbio Science & Technology Co., Ltd. (Beijing, China). Chromatographic grade methanol, acetonitrile, and formic acid were obtained from Merck KGaA (Darmstadt, Germany). The Compound Danshen Dropping Pills were sourced from Tably Pharmaceutical Group Co., Ltd. (Tianjin, China). All primers were carried out by BioSune Biotechnology Co., Ltd. (Shanghai, China). Table S1 shows the other instruments, reagents, and consumables used in this experiment.

### 4.2. Plant Collection, Extraction, and Isolation

Entire plants of *L. supina* were collected in June 2016 from Tongliao city (Inner Mongolia Autonomous Region, China) and labeled XZC201606. The preparation of the ethanol extract of this plant and its four differential fractions was described in detail in our previous studies [22]. In brief, entire plants were air-dried and crushed, yielding a total of 38.0 kg. They were then treated with 95% ethanol–water followed by 50% ethanol–water at room temperature through exhaustive percolation. The solvents were subsequently removed under reduced pressure at 60 °C, producing 8.7 kg of a black viscous extract designated LS, with an extraction yield of 22.90%. The LS was suspended in 8 L of water and then treated five times with an equal volume of petroleum ether. This produced a petroleum ether fraction (LSA, 1320 g) along with the residual aqueous fraction. The latter fraction was subsequently concentrated under reduced pressure at 60 °C, resuspended in water, and subjected to D101 macroporous resin CC, in which water and 30%, 60%, and 95% ethanol–water were used as eluting solvents. This process yielded a water fraction (LSB, 6148 g), 30% ethanol–water fraction (LSC, 669 g), 60% ethanol–water fraction (LSD, 260 g), and 95% ethanol–water fraction (145 g, combined with LSA).

The LSD fraction (235.0 g) was subjected to silica gel CC, which was eluted with dichloromethane–methanol mixtures (CH_2_Cl_2_–CH_3_OH) at ratios of 30:1, 1:40, and 0:100 (*v*/*v*), yielding fractions of LSD1 (40.0 g), LSD2 (137.0 g), and LSD3 (52.0 g) in sequence, respectively. Further separation of LSD1 was performed by silica gel CC with a CH_2_Cl_2_–CH_3_OH gradient (100:1, 50:1, 30:1, 3:1, and 1:1, *v*/*v*). After reducing the pressure and combining the fractions via thin-layer chromatography, seven subfractions (LSD1-0 to LSD1-6) were obtained. The LSD1-0 subfraction (130.0 mg) was then subjected to Sephadex LH-20 CC using CH_3_OH, resulting in Number **30** (7.0 mg, 0.3 µg/mg of LSD).

The LSD1-2 fraction (12.34 g) was separated via ODS MPLC via a CH_3_OH–H_2_O gradient system (30%, 40%, 50%, 60%, 70%, and 100%, *v*/*v*), yielding eight subfractions (LSD1-2-1 to LSD1-2-8). Among them, the LSD1-2-3 fraction (0.54 g) was further purified by recrystallization and then employed preparative HPLC within a 10% acetonitrile–water (CH_3_CN–H_2_O) solution containing 0.1% formic acid, resulting in yields of Numbers **28** (144.5 mg, 6.1 µg/mg of LSD) and **29** (94.2 mg, 4.0 µg/mg of LSD). Moreover, the LSD1-2-4 fraction (2.60 g) was processed through Sephadex LH-20 CC using CH_3_OH as the eluent, resulting in seven subfractions (LSD1-2-41 to LSD1-2-47). Recrystallization of LSD1-2-45 (486.3 mg) yielded Number **16** (138.1 mg, 5.9 µg/mg of LSD). Additionally, the LSD1-2-5 fraction (2.67 g) was separated by silica gel CC using a CH_2_Cl_2_–CH_3_OH system (100:0, 100:1, 50:1, and 20:1, *v*/*v*), resulting in seven subfractions (LSD1-2-51 to LSD1-2-57). Furthermore, recrystallization of LSD1-2-56 (2.12 g) resulted in Number **18** (45.8 mg, 1,9 µg/mg of LSD).

Fraction LSD1-3 (5.38 g) was subjected to silica gel CC via a CH_2_Cl_2_–CH_3_OH gradient (200:1, 80:1, 50:1, 30:1, and 10:1, *v*/*v*), resulting in nine fractions (LSD1-3-1 to LSD1-3-9). Fraction LSD1-3-8 (1.97 g) was further separated via ODS MPLC with a CH_3_OH–H_2_O gradient (20%, 30%, 45%, 50%, 65%, 80%, and 90%, *v*/*v*), yielding 15 subfractions (LSD1-3-8-1 to LSD1-3-8-15). Number **15** (68.4 mg, 2.9 µg/mg of LSD) was isolated from LSD1-3-8-5 after recrystallization and subsequent purification via preparative HPLC (22% CH_3_CN–H_2_O containing 0.1% formic acid).

Fraction LSD1-6-1 (1.81 g) was processed through ODS MPLC, eluted with CH_3_OH–H_2_O (40%, 60%, and 100%, *v*/*v*), and resulted in eight fractions (LSD1-6-11 to LSD1-6-18). Among them, Fraction LSD1-6-16 (0.43 g) was further purified by preparative HPLC (70% CH_3_OH–H_2_O) to obtain Numbers **17** (40.3 mg, 1.7 µg/mg of LSD) and **19** (212.0 mg, 9.0 µg/mg of LSD).

Fraction LSD1-6-2 (1.67 g) was separated via Sephadex LH-20 CC with CH_3_OH as the eluent, resulting in three fractions (LSD1-6-21 to LSD1-6-23). Fraction LSD1-6-23 (0.77 g) was then subjected to silica gel CC with a CH_2_Cl_2_–CH_3_OH gradient (100:0, 100:1, 50:1, 30:1, 10:1, and 5:1, *v*/*v*), producing two fractions, namely, LSD1-6-231 and LSD1-6-232. Afterward, Numbers **6** (4.5 mg, 0.2 µg/mg of LSD) and **9** (4.0 mg, 0.2 µg/mg of LSD) were obtained via preparative HPLC (50% CH_3_OH–H_2_O) from the LSD1-6-232 fraction.

Fraction LSD1-6-3 (1.51 g) was fractionated by Sephadex LH-20 CC with CH_3_OH as the eluent, yielding seven subfractions (LSD1-6-31 to LSD1-6-37). Fraction LSD1-6-35 (138.0 mg) was further purified via preparative HPLC (45% CH_3_CN–H_2_O) to isolate Numbers **4** (16.8 mg, 0.7 µg/mg of LSD) and **7** (39.3 mg, 1.7 µg/mg of LSD).

Fraction LSD2 (137.0 g) was subjected to silica gel CC using a CH_2_Cl_2_–CH_3_OH gradient (50:1, 10:1, and 1:5, *v*/*v*). After solvent removal under reduced pressure, three fractions were obtained (LSD2-1 to LSD2-3). Fraction LSD2-1 (50.16 g) underwent MCI CC with a CH_3_OH–H_2_O gradient (water, 20%, 40%, 60%, 80%, and 90%, *v*/*v*), producing six fractions, namely, LSD2-1-1 to LSD2-1-6. Fraction LSD2-1-6 (8.85 g) was further separated by Sephadex LH-20 CC with CH_3_OH as the eluent, yielding seven fractions (LSD2-1-6-1 to LSD2-1-6-7). From LSD2-1-6-1 (0.38 g), Number **21** (249.0 mg, 10.6 µg/mg of LSD) was obtained via preparative HPLC with 30% CH_3_CN–H_2_O containing 0.1% formic acid as the solvent. Fraction LSD2-1-6-6 (3.42 g) was processed through Sephadex LH-20 CC with CH_3_OH used as the eluent, resulting in three subfractions (LSD2-1-6-61 to LSD2-1-6-63). In addition, Subfraction LSD2-1-6-62 (197.0 mg) yielded Numbers **21** (4.1 mg,0.2 µg/mg of LSD) and **27** (96.4 mg, 4.1 µg/mg of LSD) after purification by preparative HPLC (65% CH_3_OH–H_2_O). Afterward, Subfraction LSD2-1-6-63 (203.0 mg) was processed via semipreparative HPLC (47% CH_3_CN–H_2_O) to isolate Numbers **8** (6.2 mg, 0.3 µg/mg of LSD) and **22** (145.7 mg, 6.2 µg/mg of LSD).

Fraction LSD2-2 (50.16 g) underwent MCI CC via a CH_3_OH–H_2_O gradient (water, 20%, 40%, 60%, 80%, and 90%, *v*/*v*), yielding 12 fractions (LSD2-2-1 to LSD2-2-12), along with Numbers **21** (57.6 mg, 2.5 µg/mg of LSD) and **23** (231.4 mg, 9.8 µg/mg of LSD). Fraction LSD2-2-7 (3.58 g) was further separated by Sephadex LH-20 CC with CH_3_OH as the eluent, resulting in eight fractions (LSD2-2-7-1 to LSD2-2-7-8). From LSD2-2-7-3 (122.0 mg), Number **3** (30.2 mg, 1.3 µg/mg of LSD) was isolated via preparative HPLC with 25% CH_3_CN–H_2_O containing 0.1% formic acid as the solvent. Additionally, Fraction LSD2-2-7-6 (0.36 g) was subjected to preparative HPLC (22% CH_3_CN–H_2_O containing 0.1% formic acid), yielding Numbers **13** (26.5 mg, 1.1 µg/mg of LSD) and **14** (172.1 mg, 7.3 µg/mg of LSD).

Fraction LSD2-2-8 (9.80 g) was separated by silica gel CC using a CH_2_Cl_2_–CH_3_OH gradient (16:1, 14:1, 10:1, 8:1, 6:1, and 3:1, *v*/*v*) with solvent recovery under reduced pressure. It was then combined by thin-layer chromatography into 13 subfractions (LSD2-2-8-1 to LSD2-2-8-13). Fraction LSD2-2-8-6 (4.74 g) was further separated by Sephadex LH-20 CC using CH_3_OH as the eluent, producing five subfractions (LSD2-2-8-6-1 to LSD2-2-8-6-5). As a result, Number **27** (825.7 mg, 35.1 µg/mg of LSD) was isolated from the LSD2-2-8-6-5 subfraction (1.35 g) via preparative HPLC (43% CH_3_CN–H_2_O).

Subfractions LSD2-2-8-7 (0.46 g) and LSD2-2-8-11 (0.57 g) were recrystallized to obtain Numbers **10** (41.8 mg, 1.8 µg/mg of LSD) and **11** (272.3 mg, 11.6 µg/mg of LSD), respectively. Subfraction LSD2-2-8-9 (1.97 g) was separated by Sephadex LH-20 CC with CH_3_OH as the eluent, resulting in six subfractions (LSD2-2-8-9-1 to LSD2-2-8-9-6). From LSD2-2-8-9-2 (0.77 g), Numbers **1** (23.8 mg, 1.0 µg/mg of LSD) and **2** (514.8 mg, 21.9 µg/mg of LSD) were isolated by preparative HPLC using CH_3_CN–H_2_O (22:78, *v*/*v*) as the eluent.

Fraction LSD2-2-9 (4.69 g) was subjected to Sephadex LH-20 CC using CH_3_OH as the eluent, yielding nine fractions (LSD2-2-9-1 to LSD2-2-9-9). Subfraction LSD2-2-9-7 (0.56 g) was subsequently purified via preparative HPLC with a 26% CH_3_CN–H_2_O system, resulting in the isolation of Numbers **25** (348.4 mg, 14.8 µg/mg of LSD) and **26** (45.2 mg, 1.9 µg/mg of LSD). Subfraction LSD2-2-9-8 (32.0 mg) was further separated by semipreparative HPLC with a 33% CH_3_CN–H_2_O mixture, yielding Number **5** (9.7 mg, 0.4 µg/mg of LSD). Fraction LSD2-2-10 (10.25 g) was purified on a silica gel column using a CH_2_Cl_2_–CH_3_OH gradient (20:1, 16:1, 12:1, and 5:1, *v*/*v*). Solvent removal under reduced pressure afforded eight fractions (LSD2-2-10-1 to LSD2-2-10-8). Subfraction LSD2-2-10-8 (6.04 g) was eluted with CH_3_OH and separated by Sephadex LH-20 CC into four fractions (LSD2-2-10-8-1 to LSD2-2-10-8-4). Further recrystallization of Subfraction LSD2-2-10-8-3 yielded two subfractions, LSD2-2-10-8-31 and LSD2-2-10-8-32. Among them, Subfraction LSD2-2-10-8-31 was purified by preparative HPLC using a 37% CH_3_CN–H_2_O mixture, isolating Numbers **20** (245.9 mg, 10.5 µg/mg of LSD) and **24** (74.9 mg, 3.2 µg/mg of LSD). On the other hand, Subfraction LSD2-2-10-8-32 was subjected to Sephadex LH-20 CC using CH_3_OH as the eluent, resulting in five subfractions (LSD2-2-10-8-321 to LSD2-2-10-8-325). Finally, subfraction LSD2-2-10-8-324 was purified via preparative HPLC with a 50% CH_3_OH–H_2_O mixture containing 0.1% formic acid, yielding Numbers **10** (49.8 mg, 2.1 µg/mg of LSD) and **12** (50.5 mg, 2.1 µg/mg of LSD).

**Figure 9:**
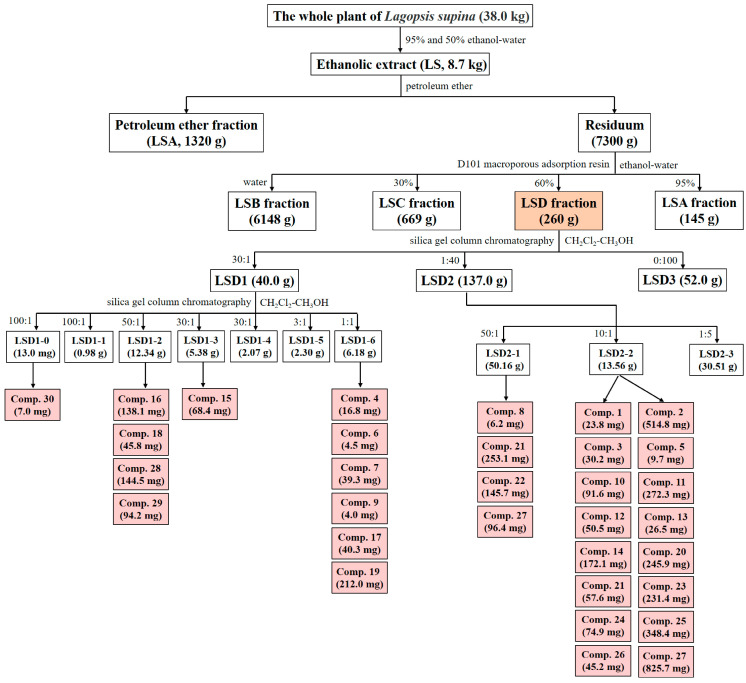
Separation process of LSD. Light orange represents the separated fraction LSD and its weight in this article, while light red represents the separated compound and its weight.

Overall, a total of 30 compounds (Nos. **1–30**) were isolated from the LSD fraction. The detailed separation process is illustrated in Figure 9.

### 4.3. Ingredient Identification

The composition was analyzed using an HPLC column on an Agilent 1260 Infinity II LC system. The mobile phase included acetonitrile (A) and aqueous solution (B), both of which were supplemented with 0.1% formic acid. The following solvent gradient was used: 25–31% A (0–9 min), 31–32% A (9–12 min), 32% A (12–28 min), 32–38% A (28–32 min), 38–50% A (32–53 min), 50–65% A (53–60 min), 65–100% A (60–65 min), and 100% A (65–75 min).

### 4.4. Compound Identification

All the isolated compounds were identified via NMR, including ^1^H and ^13^C NMR, on Bruker Avance-400 and Bruker-600 spectrometers (Bruker, Switzerland). Moreover, the NMR data of all the compounds were confirmed by comparison with published information.

### 4.5. Zebrafish Preparation

The transgenic zebrafish line Tg (*cmlc2: EGFP*), which expresses enhanced green fluorescent protein in the heart, was provided by the Engineering Research Center of Zebrafish Models for Human Diseases and Drug Screening of Shandong Province. Embryos were derived through natural spawning and incubated at 28 °C. The feeding management of zebrafish met the requirements of the Zebrafish Book [69]. All zebrafish experiments in this study complied with the standard ethical guidelines and were ratified by the Experimental Animal Welfare Ethics Committee of the Biology Institute of Shandong Academy of Science. The ethics permit number is SWS20231229.

The day before mating, healthy adult zebrafish were placed in an ovulation box with a male-to-female ratio of 2:2. Mating occurred the following day at 8:00, and the embryos were collected 2 hours later. After being washed 3 times with water, the embryos were transferred to a light incubator and cultured with fish water containing 0.1% methylene blue at 28 °C.

### 4.6. Anti-MI Assay

MI in zebrafish was induced by ISO [15]. Healthy zebrafish larvae were sorted onto culture plates (ten embryos per pore) at 72 hours post fertilization (hpf) and split into four groups at random: the control group (C), model group (ISO), positive control group, and sample treatment group. The control group was treated with culture water, and the other groups were added to the model drug ISO (5 µg/mL) to induce MI. The positive control group also added with CDDP (250 µg/mL). The different samples were added with different concentrations in the corresponding treatment groups (25, 50, and 100 µg/mL for fractions; 25, 50, and 100 µM for compounds). To avoid the influence of pigment deposition on the results, 3% 1-Phenyl-2-thiourea was added to each group. All the groups were subsequently placed in a light incubator at 28 ± 5 °C and cultured in the dark for 5 hours. After incubation, zebrafish embryos were viewed with a fluorescence microscope, and measurements of the ventricle′s area and short axis length were taken. The stroke volume, ejection fraction, and ventricular short-axis systolic rate of the zebrafish were subsequently calculated. All the treatments were performed in triplicate. The calculation formulas for each indicator are as follows:

Storke output = V_diastole_ − V_systole_ (1)

Ejection fraction = Storke output/V_diastole_ × 100% (2)

Ventricular short axis systolic rate = [(L_diastole_ − L_systole_)/L_diastole_] × 100% (3)

L: Short axis length of the ventricle; V: Ventricular volume.

### 4.7. Molecular Docking Analysis

The detailed procedure can be found in our previous studies [21]. Molecular docking between Number **25** and its potential target proteins, including KDR, PI3K, Akt, MAPK, Bcl-2, Bax, and Caspase3, was performed via AutoDock Vina 1.1.2. Additionally, ERK and p38 are two forms of MAPK [70,71,72]. The PDB codes for KDR, PI3K, Akt, Erk, p38, Bcl-2, Bax and Caspase3 are 1YWN [73], 6AUD [74], 4GV1 [75], 4QTB [76], 1A9U [77], 6O0L [78], 4S0O [79], and 5I9B [80], respectively.

### 4.8. RT-qPCR Assay

Except for with 30 zebrafish in each pore, the experimental methods were the same as those described in the Section 4.6. Zebrafish from each group were collected separately. Total RNA was extracted from fresh fish via the FastPure Cell/Tissue Total RNA Isolation Kit-BOX 2. cDNA was synthesized via HiScript^®^ III RT SuperMix for qPCR (+gDNA wiper). The expression levels of the genes kdr, pik3cb, akt2, mapk1, mapk11, mapk14, bcl-2b, bax, and caspase3 were identified via the ChamQ Universal SYBR qPCR Master Mix. A LightCyler 96 system (Roche, Basel, Switzerland) was used for RT-qPCR experiments; the reaction conditions were as follows: 30 s at 95 °C, followed by 40 cycles of 95 °C for 15 s and 60 °C for 30 s. Rpl13a served as an internal control. Each PCR test was carried out in four replicates. The relative quantity of transcripts was estimated via the 2^−△△Ct^ equation. Table S2 shows the sequences of all the primers used.

### 4.9. Statistical Analysis

Statistical significance was analyzed by one-way analysis of variance for two or more groups via GraphPad Prism software (9.2.3). *p* < 0.05 was considered statistically significant.

## 5. Conclusions

In this pioneering study, LSD was the active fraction responsible for the anti-MI effect of *L. supina*, and 30 phytochemicals were obtained and identified from LSD. Lignans and flavonoids were recognized as the primary pharmacological components of LSD among these compounds. Notably, tiliroside was selected as the active compound for further research of its mechanism of action. This is also the first discovery of anti-MI activity for tiliroside. Gene expression analysis and molecular docking results indicated that tiliroside exerted anti-MI activity by activating the *kdr*-mediated PI3K-Akt and MAPK pathways. This work provides insight into the chemical composition of LSD from *L. supina*, together with the pharmacodynamics and potential mechanisms of tiliroside in treating MI.

## Data Availability

The original contributions presented in this study are included in the article/Supplementary Materials. Further inquiries can be directed to the corresponding author(s).

## Supplementary Materials

The supporting information can be downloaded at: https://www.mdpi.com/article/10.3390/ijms26052313/s1.

## Abbreviations

The following abbreviations are used in this manuscript:

MI	Myocardial ischemia
LS	The ethanol extract of *L. supina*
LSD	The 60% ethanol–water extract from macroporous adsorbed resin
ISO	Isoprenaline hydrochloride
CDDP	Compound Danshen Dropping Pills
hpf	Hours past fertilization
TCM	Traditional Chinese medicine
CC	Column chromatography
ODS	Octadecylsilyl
