# Supplementary material for: Tiliroside from Lagopsis supina Ameliorates Myocardial Ischemia Injury in Zebrafish by Activating the kdr-Mediated PI3K-Akt and MAPK Signaling Pathways
Source: Int J Mol Sci. 2025 Mar 5;26(5):2313. doi: 10.3390/ijms26052313 (PMC11900366; doi:10.3390/ijms26052313)
Supplement: Supplementary file 1 [file ijms-26-02313-s001.zip › ijms-3484596-supplementary.pdf]

Tiliroside from *Lagopsis supina* ameliorate myocardial ischemia injury in zebrafish through activating *kdr*-mediated PI3K-Akt and MAPK signaling pathways

Yuqing Dong [1,†], Xiaoyi Xia [2,†], Miaoyunhuan Wang [1], Jiahao Yu [1], Lizhen Wang [1], Li Yang [2], Kechun Liu [1], Junwei He [2,*], Xiaobin Li [1,*]

[1] Engineering Research Center of Zebrafish Models for Human Diseases and Drug Screening of Shandong Province, Biology Institute, Qilu University of Technology (Shandong Academy of Sciences), Jinan 250103, China

[2] Jiangxi University of Chinese Medicine, Nanchang 330004, China

† These authors contributed equally to this work.

*Corresponding author:

Xiaobin Li, 28789 East Jingshi Road, Jinan, Shandong Province, China, Tel: +86-531-82605331, E-mail address: lixb@sdas.org.

Junwei He, E-mail address: hjwjn2008@163.com.

Table of contents

Table S1 Instruments, reagents, and consumables used in this experiment 1

Table S2 Primer sequences used for RT-qPCR experiments 3

Table S3 The information of 30 phytochemicals from the LSD fraction 4

Table S1 Instruments, reagents, and consumables used in this experiment.

Catalogue	Supplier
1-Phenyl-2-thiourea	Shanghai McLean Biochemical Technology Co., Ltd. (Shanghai, China)
FastPure Cell/Tissue Total RNA Isolation Kit-BOX 2 (RC101-01)	
HiScript® III RT SuperMix for qPCR (+gDNA wiper) (R323-01)	Vazyme Biotech Co., Ltd. (Nanjing, China)
ChamQ Universal SYBR qPCR Master Mix (Q711-02)	
LC-20AT HPLC	Shimadzu Co., Ltd. (Japan)
LC-6AD preparative HPLC	Shimadzu Co., Ltd. (Japan)
AV-600 NMR	Bruker Co., Ltd. (Germany)
CP214 electronic analytical balance	Shanghai Ohaus instruments Co., Ltd. (China)
Three-purpose UV analyzer	Shanghai Anting electronic instrument factory (China)
Rotavapor R220SE	BUCHI Co., Ltd. (Germany)
Rotary evaporator	EYELA Co., Ltd. (Japan)
DHG-Ⅲ electrothermal blowing dry box	Shanghai Xinmiao Medical Device Manufacturing Co., Ltd. (China)
Analytical grade methanol, ethanol, methylene chloride, petroleum ether, and ethyl	Xilong Chemical Co., Ltd. (China)

acetate	
Chromatographic grade methanol, acetonitrile, and formic acid	American World Co., Ltd. (USA)
Thin layer chromatography silica gel G254	Qingdao ocean Chemical Co., Ltd. (China)
D101 macroporous resin	Xi'An Sunresin New Materials Co., Ltd. (China)
Column chromatography silica gel	Qingdao ocean Chemical Co., Ltd. (China)
HPLC column (InertSustain C18, 5 μm, 4.6 × 250 mm)	SHIMADZU Co., Ltd. (Japan)
Semi-preparative HPLC column (5 μm, 10 × 250 mm）	YMC Co., Ltd. (Japan)
preparative HPLC column (YMC-Pack ODS-A, 5 μm, 20 × 250 mm)	YMC Co., Ltd. (Japan)

Table S2 Primer sequences used for RT-qPCR experiments.

Gene	Primer	Sequence (5'→3')
kdr	Forward	GTGGTCAACCTTCTAGGGGC
	Reverse	GCGTCCGTTTCTTGTAGGGA
pik3cb	Forward	GGAGGCGCAGACATATCCTC
	Reverse	AGAACAGGCAGGAATGGTCG
akt2	Forward	AAGCTCGTTCCACCCTTCAA
	Reverse	TGTGTGCGTGTATCTGGGTC
mapk1	Forward	CGCTTACGGCATGGTTTGTT
	Reverse	TACGTCTGGTGCTCAAACGG
mapk11	Forward	GAGGTGCCAGAACGATACCAG
	Reverse	GTGGATGAGGGACTGGAAAGG
mapk14	Forward	AGGGCCTGAGCTCTTGATGAAAAT
	Reverse	TGTGTCCAGAACCAGCATCTT
bcl-2b	Forward	TCATAACGTGCTACGGGAGG
	Reverse	GTGCCCCCAAACTCGAAAAA
bax	Forward	TACTTTGCCTGTCGCCTTGT
	Reverse	AGCGAGGAAAACTCCGACTG
caspase3	Forward	CTTTGATCGCAGGACAGGCA
	Reverse	GTGATCGTCATGGGCAACTG
rpl13a	Forward	TCTGGAGGACTGTAAGAGGTATGC
	Reverse	AGACGCACAATCTTGAGAGCAG

Table S3 The information of 30 phytochemicals from the LSD fraction.

No.	Name	Molecular formula	Molecular weight	CAS number
1	compound 3	$C_{36}H_{48}O_{19}$	784.76	159354-70-8
2	stachysoside D	$C_{36}H_{48}O_{19}$	784.76	135010-56-9
3	artselaeroside B	$C_{37}H_{50}O_{20}$	814.78	220381-77-1
4	apigenin	$C_{15}H_{10}O_5$	270.24	520-36-5
5	luteolin	$C_{15}H_{10}O_6$	286.24	491-70-3
6	chrysoeriol	$C_{16}H_{12}O_6$	300.27	491-71-4
7	kaempferol	$C_{15}H_{10}O_6$	286.24	520-18-3
8	quercetin	$C_{15}H_{10}O_7$	302.24	117-39-5
9	isorhamnetin	$C_{16}H_{12}O_7$	316.27	480-19-3
10	apigenin-7-O-*β*-D-glucopyranoside	$C_{21}H_{20}O_{10}$	432.38	578-74-5
11	rhoifolin	$C_{27}H_{30}O_{14}$	578.52	17306-46-6
12	kaempferol-3-O-*β*-D-glucopyranoside	$C_{21}H_{20}O_{11}$	448.38	480-10-4
13	isorhamnetin-3-O-*β*-D-[6''-(3-hydroxy-3-methylglutaryl)]-O-*β*-D-glucoside	$C_{28}H_{30}O_{16}$	622.53	939403-65-3
14	kaempferol-3-O-*β*-D-glucopyranoside-6''-(3-hydroxy-3-methylglutarate	$C_{27}H_{28}O_{15}$	592.51	157407-84-6
15	*p*-coumaric acid	$C_9H_8O_3$	164.16	7400-08-0
16	ferulic acid	$C_{10}H_{10}O_4$	194.19	1135-24-6
17	*cis*-*p*-hydroxyl ethyl cinnamate	$C_{11}H_{12}O_3$	192.21	7361-92-4
18	caffeic acid ethylester	$C_{11}H_{12}O_4$	208.21	102-37-4
19	*trans*-*p*-hydroxyl ethyl cinnamate	$C_{11}H_{12}O_3$	192.21	7362-39-2
20	apigenin-7-O-(6''-(*E*)-*p*-coumaroyl)-*β*-D-galactopyranoside	$C_{30}H_{26}O_{12}$	578.53	480453-57-4
21	apigenin-7-O-(6''-*E*-*p*-coumaroyl)-*β*-D-glucopyranoside	$C_{30}H_{26}O_{12}$	578.53	105815-90-5
22	apigenin-7-O-(3'',6''-*di*-(*E*)-*p*-coumaroyl)-*β*-D-galactopyranoside	$C_{39}H_{32}O_{14}$	724.67	480990-58-7
23	apigenin-7-O-(3''-*p*-coumaryl)-glucoside	$C_{30}H_{26}O_{12}$	578.53	171367-93-4
24	palhinoside A	$C_{60}H_{52}O_{24}$	1157.05	2697157-15-4
25	tiliroside	$C_{30}H_{26}O_{13}$	494.53	20316-62-5

26	*cis*-tiliroside	$C_{30}H_{26}O_{13}$	494.53	163956-16-9
27	anisofolin A	$C_{39}H_{32}O_{14}$	724.67	83529-71-9
28	vanillic acid	$C_8H_8O_4$	168.15	121-34-6
29	syringic acid	$C_9H_{10}O_5$	198.17	530-57-4
30	4-(9H-B-carbolin-1-yl)-4-oxobut-2-enoic acid methyl ester	$C_{16}H_{12}N_2O_3$	280.28	1058724-59-6
